# Chemical Transformations of Benzyl Alcohol Caused by Atomic Chlorine

**DOI:** 10.3390/molecules29133124

**Published:** 2024-06-30

**Authors:** Dariusz S. Sarzyński, Irena Majerz

**Affiliations:** Department of Basic Chemical Sciences, Faculty of Pharmacy, Wroclaw Medical University, Ul. Borowska 211A, 50-556 Wrocław, Poland; majerz@yahoo.com

**Keywords:** chlorine atoms, benzyl alcohol, UV radiation, polluted troposphere, GC, mass spectrometry

## Abstract

Atomic chlorine present in the polluted troposphere can form potentially carcinogenic compounds as a result of a reaction with a natural product. This paper examines the stability of benzyl alcohol—a natural product commonly found in cosmetics—in interaction with atomic chlorine, which is becoming ever more present in the Earth’s atmosphere as a result of its pollution. Research shows that atomic chlorine generated in the gas phase easily penetrates the liquid phase of benzyl alcohol, resulting in the formation of hydrochloric acid. The resulting HCl initiates further transformations of benzyl alcohol. Our study presents the amounts of the reaction products. The quantitative analysis was made using the GC method, and all the products were identified using the GC-MS method. The products include dichloromethyl benzene, 2-chlorobenzyl alcohol, and 3-chlorobenzyl alcohol, which are harmful, but are formed in very small amounts. The harmful substance occurring in a much higher amount is benzyl chloride—that is a product of acidification of benzyl alcohol by HCl.

## 1. Introduction

Benzyl alcohol is a commonly used natural substance [1,2,3], which is considered safe in many uses [4]. Due to its characteristic smell, it is a frequent ingredient in perfumes [5]. Because it affects viscosity, it is used in creams to regulate their consistency, while it also acts as a solvent for other substances present in cosmetics. Benzyl alcohol has antibacterial and antifungal properties, and it has a stabilizing effect on cosmetics, which allows for their long-term use. Typically, benzyl alcohol can be found in shower gels and emulsions, bubble baths, body lotions and creams, as well as face and foot creams, facial and body cleansing gels, and similar products. Other products in which benzyl alcohol is used are non-cosmetic cleaning agents and detergents.

Benzyl alcohol is also a natural ingredient of a large number of food products, such as cocoa, apricots, honey, green beans, mushrooms, cranberries, cherries, grapes, chestnuts, almonds, or cloves [6]. Therefore, it is not surprising that benzyl alcohol is added to food as a preservative and a flavor or taste enhancer [7].

Although benzyl alcohol is labelled as H317 [8], which means that it can cause an allergic skin reaction, according to the Cramer classification, it is considered a safe substance, and belongs to class I, which means low toxicity, and so it may be used as an ingredient in medicines [9]. If a drug substance does not dissolve in water at a desired concentration, organic hydrophilic solvents should be utilised. For this purpose, benzyl alcohol is used in injection solutions at a concentration of less than 5%. It also acts as a solvent and preservative in topical steroid drugs, injectable drugs, such as vitamin B12, heparin, and steroids. Due to its bacteriostatic effect on Gram-positive bacteria and on yeast, mold, and fungi, benzyl alcohol in concentrations of up to 2% is used as a preservative in oral, parenteral and topical preparations. As an agent that improves the solubility of drugs that are sparingly soluble in water, it is used in intravenous, intramuscular, and topical preparations. Examples include injections of dexamethasone, atracurium besylate, or amiodarone hydrochloride [10]. Recently, attempts were been made to use benzyl alcohol as a medicinal substance [11,12,13,14,15,16].

Benzyl alcohol is relatively stable, although exposition to air causes its slow oxidization to benzaldehyde and subsequently to benzoic acid [17,18,19]. The reaction of benzyl alcohol with benzaldehyde produces benzaldehyde dibenzyl acetal, which is an impurity of benzyl alcohol [20]. Subsequently, benzaldehyde dibenzyl acetal is converted to a new compound, which was identified as benzaldehyde benzylmethyl acetal.

The amount of benzaldehyde dibenzyl acetal in benzyl alcohol indicates its trace presence and the change in the amount of this impurity over a longer period of time is not significant.

The aim of this work is to investigate the influence of atomic chlorine generated in the gas phase on benzyl alcohol molecules in the liquid phase. This reaction is important because atomic chlorine is present in the Earth’s atmosphere, especially in coastal areas [21]. Chlorine atoms are created in sea aerosol, near the surface of seas and oceans [22]. Large amounts of Cl atoms are formed as a result of the reaction of HCl (serves as a chlorine reservoir in the troposphere) with OH radicals in the coastal marine boundary layer [23]. Chlorine atoms (Cl) are produced by photolysis of molecular chlorine (Cl_2_), bromine chloride (BrCl), and nitrile chloride (ClNO_2_) in the troposphere. Chlorine atoms react with volatile organic pollutants or ozone to form chlorine oxide [24]. Chlorine (Cl) atoms have a significant influence on the chemical composition of the atmosphere. Increased levels of halogens were found to deplete the ozone layer in the stratosphere. It was shown that in the troposphere they can lead to the initiation of oxidation of ozone-forming hydrocarbons and modification of the oxidation capacity, as they affect the level of hydroxyl radicals (OH) and hydroperoxyl radicals (HO_2_). It is characteristic that Cl radicals show significant reactivity as an oxidizing agent for organic and inorganic compounds [25,26].

The reactivity of chlorine atoms was very well known for many years [27]. As part of the study, reactions of chlorine atoms with many compounds, such as: water and sulfuric acid [28], DMS [29], 1,3-butadiene [30,31], isoprene [32,33], chloroethane [34,35], alcohols [36,37,38,39], and benzene [40], were investigated.

Reactions of atomic chlorine with organic molecules aroused considerable interest and were long the subject of both experimental and theoretical research. They are also important in pharmacy because atomic chlorine present in the atmosphere can penetrate directly into creams and ointments on human skin. Benzyl alcohol is commonly found in numerous cosmetic and pharmaceutical products. Investigating its stability in the presence of very active atomic chlorine, which is becoming increasingly common in the atmosphere due to its pollution, seems to be an important issue from the perspective of human health (as we need to know if any potentially harmful chemical compounds are formed in the process).

## 2. Results and Discussion

**Figure 1:**
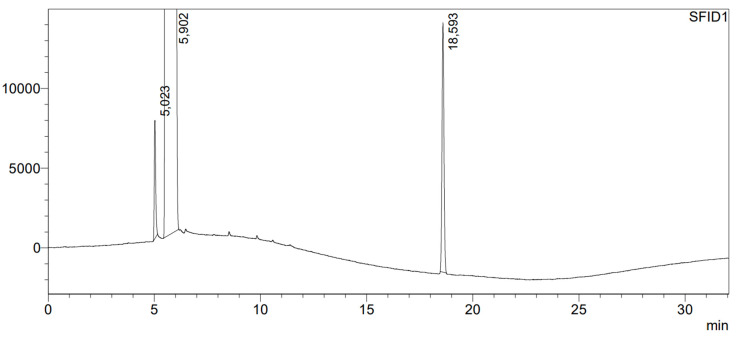
Chromatogram of benzyl alcohol from Sigma-Aldrich used in all experiments. The numbers shown in the figure represent the retention times of separated compounds, expressed in minutes.

The first stage of the research was to check for the presence of the impurities contained in the Sigma-Aldrich benzyl alcohol for synthesis used in the study. According to the chromatogram (Figure 1), the purity of the benzyl alcohol used in the later experiments is 99.86% and its significant impurities include: benzyl aldehyde in the amount of 0.04%, and benzaldehyde dibenzyl acetal in the amount of 0.10% and a retention time of 18.59 min. The testing of the purity of benzyl alcohol allowed us to conclude that it was pure enough for further study.

**Figure 2:**
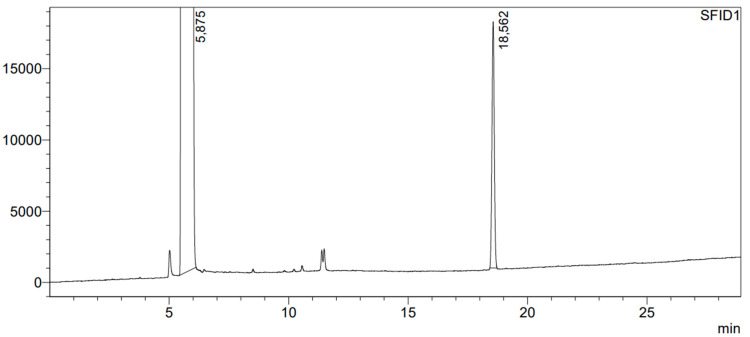
Chromatogram of benzyl alcohol after 2 h of irradiation with UV. The numbers shown in the figure represent the retention times of separated compounds, expressed in minutes.

Because atomic chlorine was generated during UV photolysis of a Cl_2_/N_2_ mixture, the stability of benzyl alcohol when subjected to UV radiation was examined. Following irradiation, the acidity of the benzyl alcohol was checked, and chromatographic separation was carried out. To perform chromatographic analysis, 1 µL of benzyl alcohol was withdrawn with a syringe and injected into the chromatograph dispenser. The chromatogram of the benzyl alcohol after irradiation is shown in Figure 2. In the chromatogram, two new peaks appeared with a retention time between 11 and 12 min, but the amount of the substances represented by these two peaks is less than 0.01%. The purity of the benzyl alcohol after photolysis amounted to 99.84%. It can be concluded that the influence of photolysis on benzyl alcohol is negligible and the products formed in very small amounts should not hinder the identification of the compounds formed as a result of the reaction of Cl with benzyl alcohol. The acidity of the benzyl alcohol after irradiation remained neutral.

**Figure 3:**
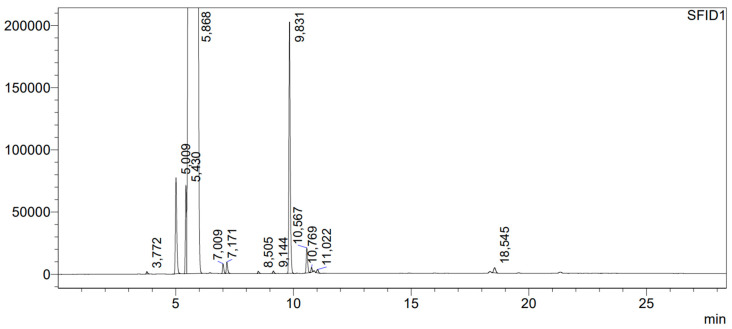
Chromatogram of benzyl alcohol after 24 h contact with the Cl_2_/N_2_ mixture. The numbers shown in the figure represent the retention times of separated compounds, expressed in minutes.

A Cl_2_/N_2_ mixture may contain trace amounts of Cl atoms, so the next experiment was designed to demonstrate whether those trace amounts of atomic chlorine cause the formation of any reaction products. For that purpose, benzyl alcohol was left in contact with a 5.09% Cl_2_/N_2_ mixture for 24 h in complete darkness. After that time, samples of the liquid phase were taken for GC and GC-MS analyses. The reaction products are presented in the chromatogram in Figure 3.

In the chromatogram, the following compounds were identified using the GC-MS method: benzyl aldehyde with a retention time of tr = 5.016 min in the amount of 0.38%, benzyl chloride—tr = 5.440 min in the amount of 0.24%, benzyl alcohol—tr = 5.859 min in the amount of 98.46%, dichloromethylbenzene—tr = 6.50 min in the amount below 0.01%; 2-chlorobenzyl alcohol—tr = 7.008 min in the amount of 0.04%, 3-chlorobenzyl alcohol—tr = 7.170 min in the amount of 0.04%, dibenzyl ether—tr = 9.827 min in the amount of 0.69%, and benzyl benzoate—tr = 10.570 min in the amount of 0.10%. After that reaction, the benzyl alcohol became acidic. The main product of the reaction was dibenzyl ether.

**Figure 4:**
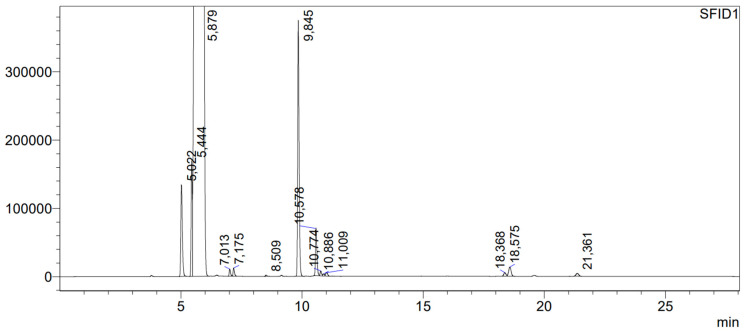
Chromatogram of benzyl alcohol after reaction with atomic chlorine generated during 3-h UV photolysis of the Cl_2_/N_2_ mixture. The numbers shown in the figure represent the retention times of separated compounds, expressed in minutes.

To achieve the aim of the test, which was to check whether atomic chlorine generated in the gas phase reacts with benzyl alcohol in the liquid phase, atomic chlorine was generated in the gas phase during UV photolysis of molecular chlorine. After three hours of irradiation, chromatographic analysis and mass spectrometry of the post-reaction mixture were performed. The chromatogram from the product separation is shown in Figure 4.

In the chromatogram (Figure 4), the following compounds were identified: benzyl aldehyde, with a retention time of tr = 5022 min in the amount of 0.64%, benzyl chloride—tr = 5444 min, in the amount of 0.62%, benzyl alcohol—tr = 5879 min, in the amount of 96.42%, dichloromethylbenzene—tr = 6.50 min, in the amount below 0.02%, 2-chlorobenzyl alcohol—tr = 7.013 min, in the amount of 0.04%, 3-chlorobenzyl alcohol—tr = 7.175 min, in the amount of 0.05%, dibenzyl ether—tr = 9.845 min, in the amount of 1.65%, and benzyl benzoate—tr = 10.774 min, in the amount of 0.31%. After the reaction, the investigated sample became acidic. The main product of the reaction was dibenzyl ether. Table 1 presents the compounds in the liquid reaction mixture.

The experiment showed that a very small content of atomic chlorine in a Cl_2_/N_2_ mixture causes the formation of small amounts of the products presented in Figure 3. Exposure of the reaction mixture to UV radiation results in the production of more atomic chlorine in the gas phase, which reacts with benzyl alcohol in the liquid phase, generating the same compounds, but in significantly larger amounts. If benzyl alcohol is left for 24 h with a mixture of 5.09% Cl_2_/N_2_ at a total pressure of 665 Torr and without access to UV radiation, its content decreases to 98.49%. After a three-hour exposure to UV radiation of benzyl alcohol in a Cl_2_/N_2_ mixture, the amount of benzyl alcohol decreased down to 96.42%, and the content of benzaldehyde increased from 0.04 to 0.64%. The main product of the reaction of benzyl alcohol with atomic Cl, which increased its amount from 0.68% to 1.65%, was dibenzyl ether. It is very characteristic that in the reaction mixture, despite the presence of benzyl alcohol and benzaldehyde, the product of their reaction, i.e., benzaldehyde dibenzyl acetal [20], was not found. The formation of 2-chlorobenzyl alcohol, 3-chlorobenzyl alcohol is associated with a simultaneous presence of atomic and molecular chlorine in benzyl alcohol. These products were not identified in the post-reaction mixture following acidification of benzyl alcohol with concentrated HCl. The probable mechanism of those reactions is that atomic chlorine abstracts the hydrogen from the benzene ring and the resulting radical reacts with Cl_2_ to form appropriate chlorobenzyl alcohol.

Dichloromethylbenzene seems to be a secondary product of the studied reaction, i.e., at the beginning, benzyl chloride is formed as a result of acidification of benzyl alcohol with HCl generated by the abstraction of hydrogen atoms from benzyl alcohol by atomic chloride. In the next step of the reaction, the chlorine atom abstracts the hydrogen atom from the CH_2_Cl group, and the resulting -C^·^HCl radical reacts with Cl_2_ to form dichloromethylbenzene.

It is very characteristic that irradiation of the Cl_2_/N_2_ mixture remaining in contact with benzyl alcohol changes its acidity from neutral to strongly acidic. The main product of the reaction of benzyl alcohol with atomic chlorine (generated in the gas phase) is dibenzyl ether. It is known that symmetrical ethers can be made from acid-catalyzed dehydration of primary alcohols. The reaction proceeds in three steps: protonation, nucleophilic substitution [S_N_2], and deprotonation [41,42,43]. The formation of dibenzyl ether suggests that the atomic chlorine generated in the gas phase after transition to liquid benzyl alcohol abstracts hydrogen atoms. As a result of the reaction, HCl is formed, which acidifies the environment and is a catalyst for the formation of dibenzyl ether.

**Figure 5:**
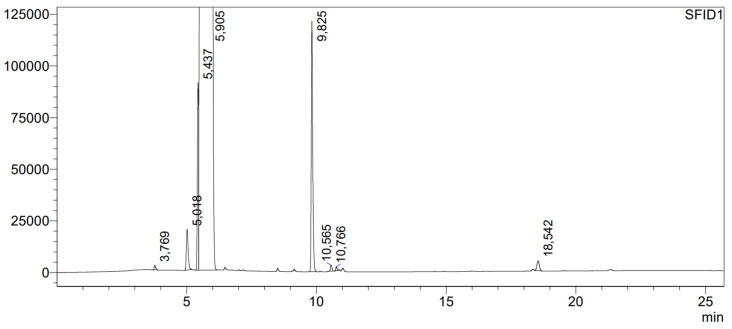
Chromatogram of benzyl alcohol after acidification with HCl. The numbers shown in the figure represent the retention times of separated compounds, expressed in minutes.

In order to check whether hydrochloric acid causes the formation of dibenzyl ether, liquid HCl was added to benzyl alcohol and left for 48 h. The chromatogram of the mixture of benzyl alcohol with HCl is shown in Figure 5. In the chromatogram, the following compounds were identified: benzyl aldehyde, with a retention time of tr = 5.018 min, in the amount of 0.09%, benzyl chloride—tr = 5.437 min, in the amount of 0.28%, benzyl alcohol—tr = 5.905 min, in the amount of 99.11%, dibenzyl ether—tr = 9.825 min in the amount of 0.47%, and benzyl benzoate—tr = 10.565 min, in the amount of 0.01%. It appears that the main reaction product is dibenzyl ether, while benzyl chloride is its second most abundant product. It is known that primary alcohols are converted into the corresponding halides as a result of nucleophilic substitution S_N_2. First, the oxygen atom is protonated, and, subsequently, the nucleophilic attack of Cl^−^ occurs. This leads to the formation of benzyl chloride, with a simultaneous elimination of the water molecule [44]. After the acidification of benzyl alcohol with HCl, no formation of 2-chlorobenzyl alcohol or 3-chlorobenzyl alcohol was observed. The products only formed during photolysis in benzyl alcohol plus the Cl_2_/N_2_ mixture and during the reaction after benzyl alcohol was left in contact with the Cl_2_/N_2_ mixture for 48 h.

After determining what chemical compounds are present in benzyl alcohol after its reaction with atomic chlorine, the question should be asked about the effect of those compounds on the human body. Benzaldehyde applied to the skin causes redness and may lead to an allergic reaction. Benzyl chloride is toxic after inhalation, oral, or dermal administration. It is very irritating to the skin, eyes, and mucous membranes. It can cause irritation of the upper respiratory tract, conjunctivitis, headaches and dizziness, weakness, increased bilirubin in the blood, and a decrease in the number of leukocytes [45]. Applied to the skin, it can cause redness and pain, and in the case of the eyes, it causes tearing, redness, pain, blurry vision, or severe burns. The International Agency for Research on Cancer classifies benzyl chloride as possibly a Category 2A or 2B human carcinogen [46]. Dichloromethylbenzene causes redness and pain of the skin and redness and pain of the eyes. In the IARC classification, it is classified as group 2A, which means that it is a probable carcinogen [47]. 2-chlorobenzyl alcohol can cause skin and eye irritation, as well as respiratory irritation. It is not included in any of the lists as a carcinogen [48]. 3-chlorobenzyl alcohol is irritating to the skin and eyes. It can also cause respiratory irritation [49]. Dibenzyl ether causes skin irritation and severe eye irritation and may also be irritating to the respiratory system. It is not classified as a carcinogen [50]. Benzyl benzoate can be absorbed through the skin and cause dryness, redness, and an allergic reaction. It causes redness of the eyes. No evidence of carcinogenicity was found. It can cross the placenta and enter the foetus [51]. Therefore, a significant amount of the reaction products of benzyl alcohol with atomic chlorine can cause multiple harmful effects on the human body.

## 3. Experimental Part

We conducted five types of experiments to study the reaction of benzyl alcohol with atomic chlorine. The first experiment focused on checking the purity of benzyl alcohol. The second type of experiments was connected with the effect of UV radiation on benzyl alcohol. The third group of experiments concentrated on the influence of the gas mixture of Cl_2_ in N_2_ on benzyl alcohol in the liquid phase. After carrying out the above preliminary experiments, the effect of atomic chlorine generated in the gas phase on liquid benzyl alcohol in the liquid phase was investigated. Additionally, the effect of liquid HCl on benzyl alcohol was checked.

Quantitative and qualitative analyses of the reaction products in the liquid phase was carried out after each experiment.

The quantitative analysis of the products was performed using the GC Shimadzu GC-2014 gas chromatograph (SHIMADZU, Kyoto, Japan) with an FID detector equipped with an Agilent Column HP-5MS UI (Santa Clara, CA, USA), length 30 m, id. 0.25 mm. The stationary layer thickness was 0.25 µm. The separation conditions were as follows: carrier gas: helium, split: 1:50, separation under constant linear velocity, pressure on the head of the column: 59.9 kPa, total flow: 58.1 mL·min^−1^, helium flow through the column: 1.08 mL·min^−1^, linear flow: 24.1 cm s^−1^. Injector temperature: 220 °C, FID temperature: 300 °C. Temperature programming: initial temperature of the column: 100 °C for 2 min, increase in the temperature: 25 °C per min, up to 250 °C. The final temperature was kept constant for 30 min. The same separation conditions were used for every separation performed on the Shimadzu chromatograph.

For the purposes of the qualitative analysis of the reaction products a GC-MS analysis was performed by means of an Agilent GC-MS 7000D (Agilent, Santa Clara, CA, USA) system. The Agilent Mass Hunter Unknown Analysis (Agilent, Santa Clara, CA, USA) program with the NIST 17 database was used for qualitative identification of the compounds. The match factor amounted to at least 90%. In order to do that, every sample was first dissolved in n-heptane at a volume proportion of 1:100.

The reagents used in the experiments included: benzyl alcohol for synthesis from Sigma-Aldrich (Merck KGaA, Darmstadt, Germany), Cl_2_ with a purity of >99.5% from Sigma-Aldrich, and N_2_ 5.0 from Linde (Kraków, Poland). A 5.09% gas mixture of Cl_2_ in N_2_ was prepared from gaseous chlorine and nitrogen.

The photolysis of benzyl alcohol, the reaction between benzyl alcohol and Cl_2_, and the reaction of atomic chlorine with benzyl alcohol were carried out in a quartz reactor. The reactor was made of a quartz tube with a diameter of 50 mm. Its total volume was 250 cm^3^. The lower part of the reactor had a semicircular shape, while the upper section was equipped with a special vacuum valve that allows the reactor to be connected to a vacuum apparatus.

In the experiments in which the photolysis of benzyl alcohol and its reactions with Cl and Cl_2_ was investigated, about 10 mL of alcohol was introduced into the reactor. The reactor was then connected to a vacuum apparatus to degas the benzyl alcohol. For degassing, the benzyl alcohol was frozen with liquid nitrogen, and while it was melting, after the liquid nitrogen was removed, the gas above the alcohol was pumped off with a vacuum pump. This procedure was repeated several times to completely remove the air dissolved in the benzyl alcohol. After complete degassing, an equilibrium between the liquid and gas phases of benzyl alcohol was established.

In the experiments in which the photolysis of benzyl alcohol and the reaction of benzyl alcohol with atomic chlorine were studied, a 150 W Osram Xe OF lamp (OSRAM, Augsburg, Germany) was used for irradiation. In the case of the photolysis of benzyl alcohol, the light from the Xe lamp was directed to illuminate only the liquid benzyl alcohol in the reactor. However, in the case of the experiments in which the influence of atomic chlorine on benzyl alcohol was studied, the radiation from the Xe lamp was directed to affect only the gas phase in the reactor.

In the experiments in which the influence of Cl_2_ on benzyl alcohol was investigated, after degassing the alcohol, a 5.09% mixture of Cl_2_ in N_2_ was introduced into the reactor. The total pressure of the gas mixture was 665 Torr. The reactor filled in this way was left for 24 h in complete darkness. After that time, the reaction products in the liquid phase were analyzed quantitatively and qualitatively. In order to study the effect of atomic chlorine on benzyl alcohol, a 5.09% mixture of Cl_2_ in N_2_ was introduced into the reactor after degassing the benzyl alcohol. The total pressure of the mixture was about 550 Torr. After filling the reactor in this way, only the gas phase was irradiated with UV radiation for 2 h. After that time, the resulting reaction products in the liquid phase were analyzed quantitatively and qualitatively.

In the last group of experiments, the direct effect of HCl on benzyl alcohol was studied. For that purpose, a few drops of concentrated hydrochloric acid were added to the degassed benzyl alcohol. The solution prepared in this way was left for 24 h in complete darkness. After that time, the composition of the liquid phase was tested quantitatively and qualitatively.

## 4. Conclusions

The reaction of benzyl alcohol with atomic chlorine results in the creation of several products which may be harmful to the human body. The only products of the investigated reaction that can be carcinogenic are dichloromethylbenzene and benzyl chloride. Dichloromethylbenzene is likely to be a secondary product of the studied reaction, i.e., at the beginning, benzyl chloride is formed as a result of acidification of benzyl alcohol with HCl. During the next step of the reaction, the chlorine atom abstracts the hydrogen atom from the $-{\mathrm{CH}}_{2}\mathrm{Cl}$ group, and the resulting $-C^{\cdot }\mathrm{HCl}$ radical reacts with Cl_2_ to form dichloromethylbenzene. It should be taken into account that the concentration of Cl_2_ in the conditions of the experiment is higher than that in the atmosphere. Therefore, the concentration of dichloromethylbenzene formed in cosmetics containing benzyl alcohol should not be high during their use.

The concentrations of the resulting 2-chlorobenzyl and 3-chlorobenzyl alcohols are also low. Although they are not secondary reaction products, their formation requires simultaneous presence of atomic and molecular chlorine in benzyl alcohol. Again, due to the lower concentration of Cl_2_ in the atmosphere in comparison to our experimental setups, the concentration of the resulting 2- and 3-chloro benzyl alcohol can be negligible.

The situation is different in the case of benzyl chloride. Benzyl chloride is the primary reaction product and is formed as a result of acidification of benzyl alcohol by HCl. It should be emphasized that the presence of Cl_2_ in the benzyl alcohol solution (i.e., also in the atmosphere) is not needed to form benzyl chloride, but in the troposphere, there are many other sources of atomic chlorine in addition to Cl_2_, which can cause a significant increase in the concentration of Cl.

However, taking into account the fact that in our studies the concentration of atomic chlorine in the gas phase was higher than that in the marine boundary layer, the benzyl alcohol content in ointments and creams does not exceed 1%, and creams may also contain antioxidants, the amounts of benzyl chloride formed should not have a harmful effect on human health.

## Figures and Tables

**Table 1:** The compounds identified in the post-reaction mixture along with their retention times and percentage yields after the reaction of benzyl alcohol with a gas mixture of Cl_2_ in N_2_ for 48 h, column A. After 3 h irradiation of Cl_2_ in the N_2_ gas phase mixture in contact with liquid benzyl alcohol, column B. After acidification of benzyl alcohol by HCl, column C.

Compound	Retention Time in min	A	B	C
Benzyl aldehyde	5.02	0.38%	0.64%	0.09%
Benzyl chloride	5.44	0.24%	0.62%	0.28%
Benzyl alcohol	5.88	98.46%	96.42%	99.11%
Dichloromethylbenzene	6.50	0.01%	0.02%	0.0%
2-chlorobenzyl alcohol	7.01	0.04%	0.04%	0.0%
3-chlorobenzyl alcohol	7.17	0.04%	0.05%	0.0%
Dibenzyl ether	9.84	0.69%	1.65%	0.47%
Benzyl benzoate	10.77	0.10%	0.31%	0.01%

## Data Availability

The original contributions presented in the study are included in the article/Supplementary Materials, further inquiries can be directed to the corresponding author.

## Supplementary Materials

The following supporting information can be downloaded at: https://www.mdpi.com/article/10.3390/molecules29133124/s1. GC-MS spectra of benzyl alcohol after reaction with atomic chlorine, generated during 3-h UV photolysis; GC-MS spectra of benzyl alcohol after 24 h of contact with a Cl2/N2 gas phase mixture.
