# Supplementary material for: Chemical Transformations of Benzyl Alcohol Caused by Atomic Chlorine
Source: Molecules. 2024 Jun 30;29(13):3124. doi: 10.3390/molecules29133124 (PMC11243310; doi:10.3390/molecules29133124)
Supplement: Supplementary file 1 [file molecules-29-03124-s001.zip › molecules-3039925-supplementary.pdf]

Unknown Analysis Report - Best Hits

Agilent Technologies

**Batch Path**	E:\AS\dane\13_04_23		
**Analysis File Name**	test.uaf		
**Analyst Name**	Admin		
**Analysis Time**	4/19/2023 10:34:49 AM		
**Data File Name**	hex21.D	**Data Path Name**	E:\AS\dane\13_04_23
**Sample Name**	hex21	**Sample Type**	Sample
**Acq Method File**	alkbenz od 100 rampa 25stna min split 200	**Acq Method Path**	E:\AS\metody\
**Acq Time**	4/13/2023 12:49:28 PM	**Operator**	
**Instrument Name**	GCMSMS	**Dilution**	1

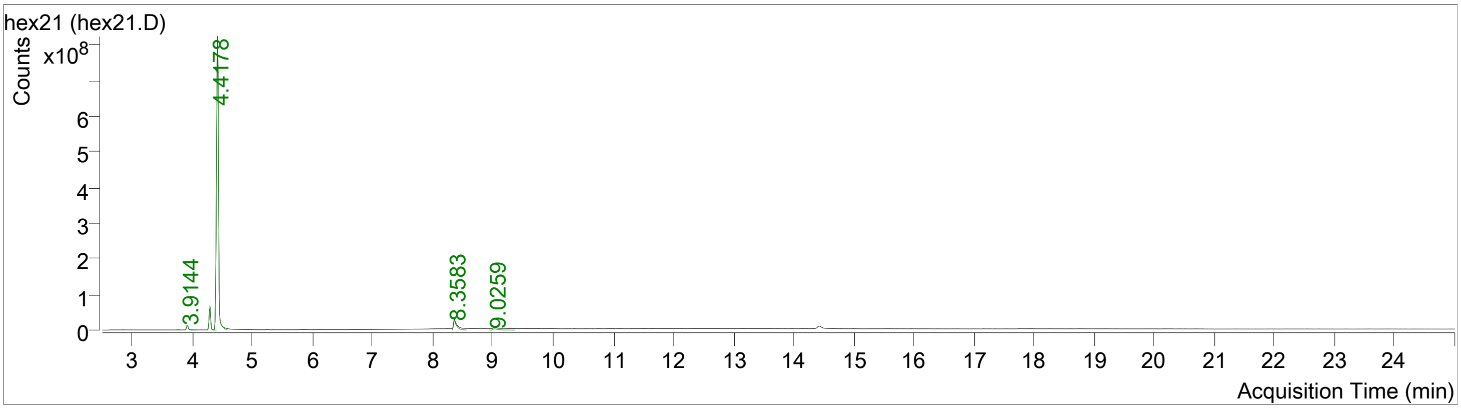

Component RT	Compound Name	CAS#	Formula	Area	Match Score	Est. Conc.
3.7648	Cyclotetrasiloxane, octamethyl-	556-67-2	C8H24O4Si4	682342	95.7	
3.9144	Benzaldehyde	100-52-7	C7H6O	25864707	96.4	
4.2899	Benzyl chloride	100-44-7	C7H7Cl	116515947	98.2	
4.4178	Benzyl alcohol	100-51-6	C7H8O	1961946901	99.6	
8.3583	Benzene, 1,1'-[oxybis(methylene)]bis-	103-50-4	C14H14O	94125837	97.5	
9.0259	Benzyl Benzoate	120-51-4	C14H12O2	20344136	93.6	

Unknown Analysis Report - Best Hits

Component RT	Compound Name	CAS#	Formula	Area	Match Score	Est. Conc.
3.7648	Cyclotetrasiloxane, octamethyl-	556-67-2	C8H24O4Si4	682342	95.7	

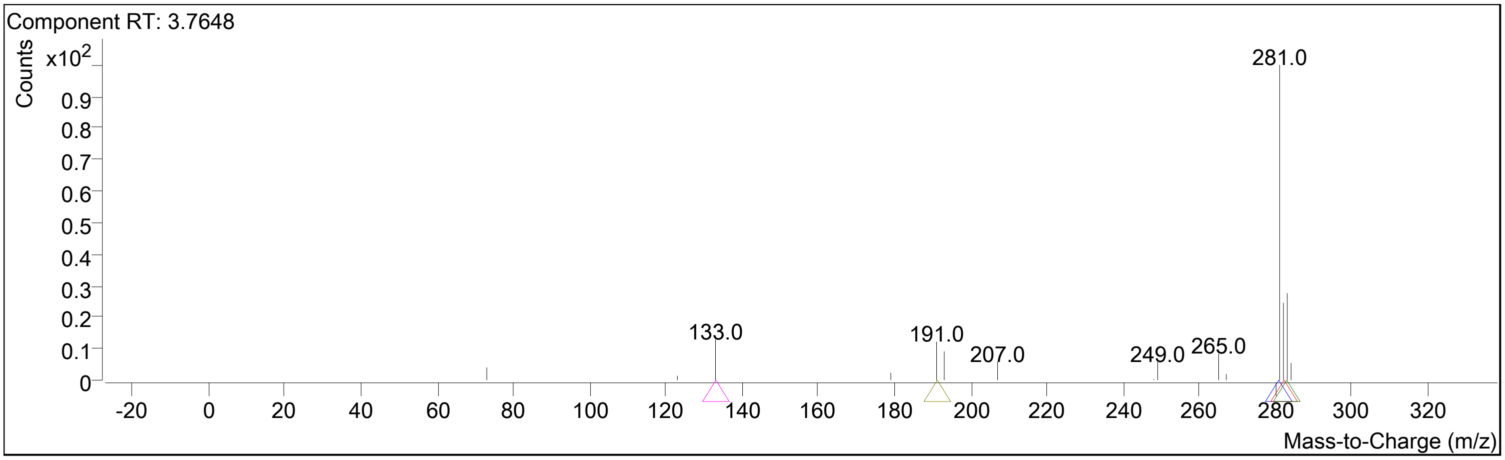

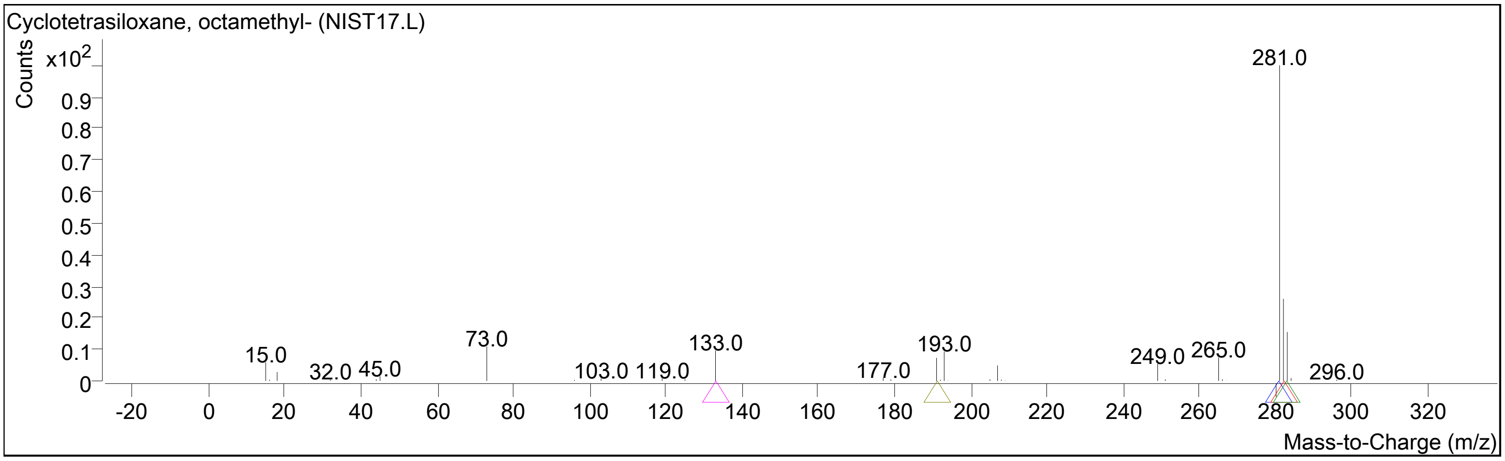

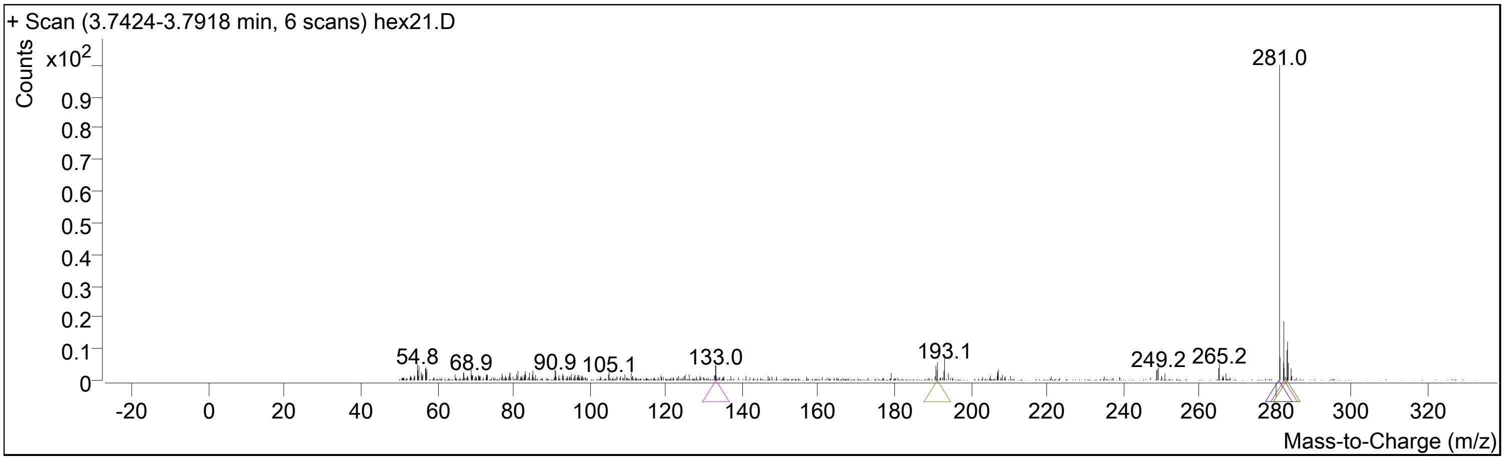

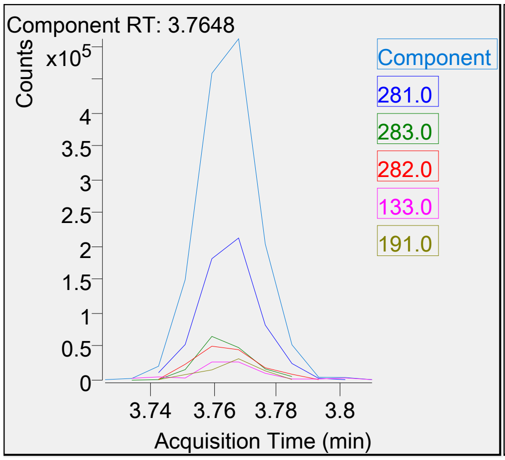

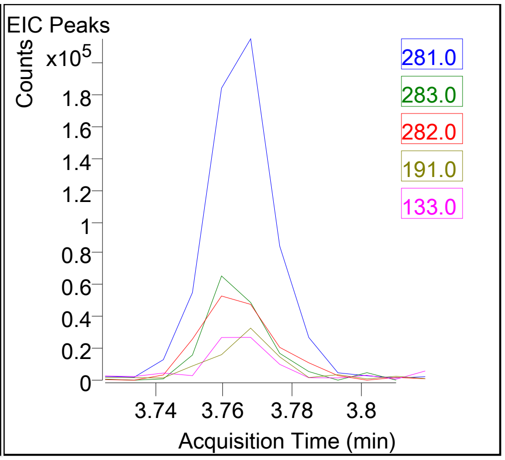

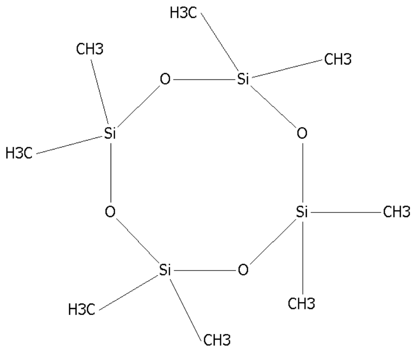

Generated at 10:36 AM on 4/19/2023

Unknown Analysis Report - Best Hits

Agilent Technologies

Component RT	Compound Name	CAS#	Formula	Area	Match Score	Est. Conc.
3.9144	Benzaldehyde	100-52-7	C7H6O	25864707	96.4	

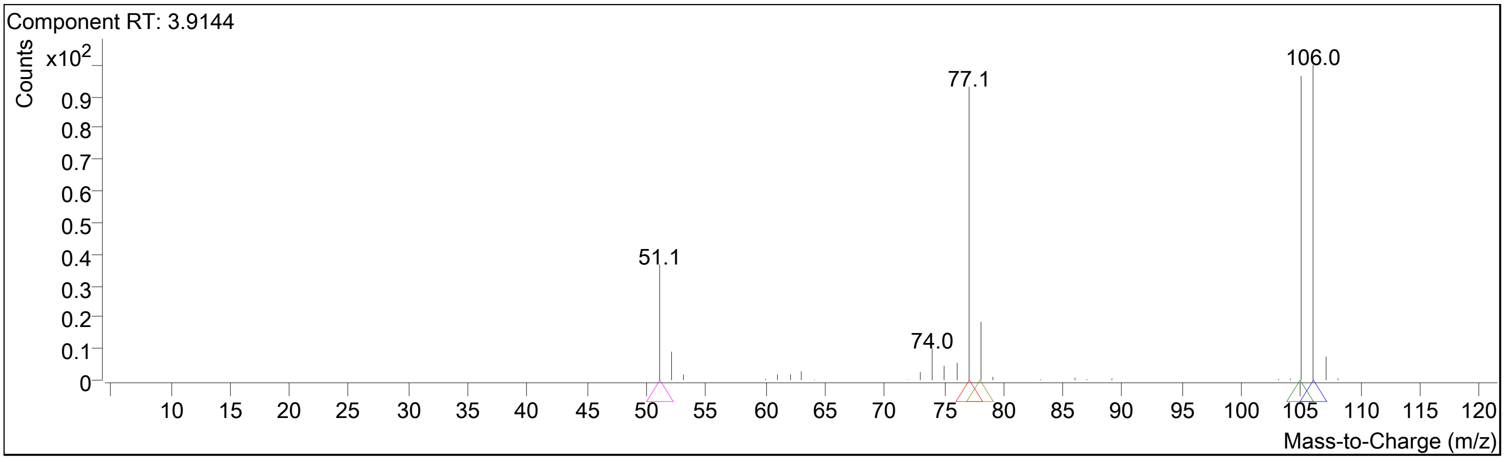

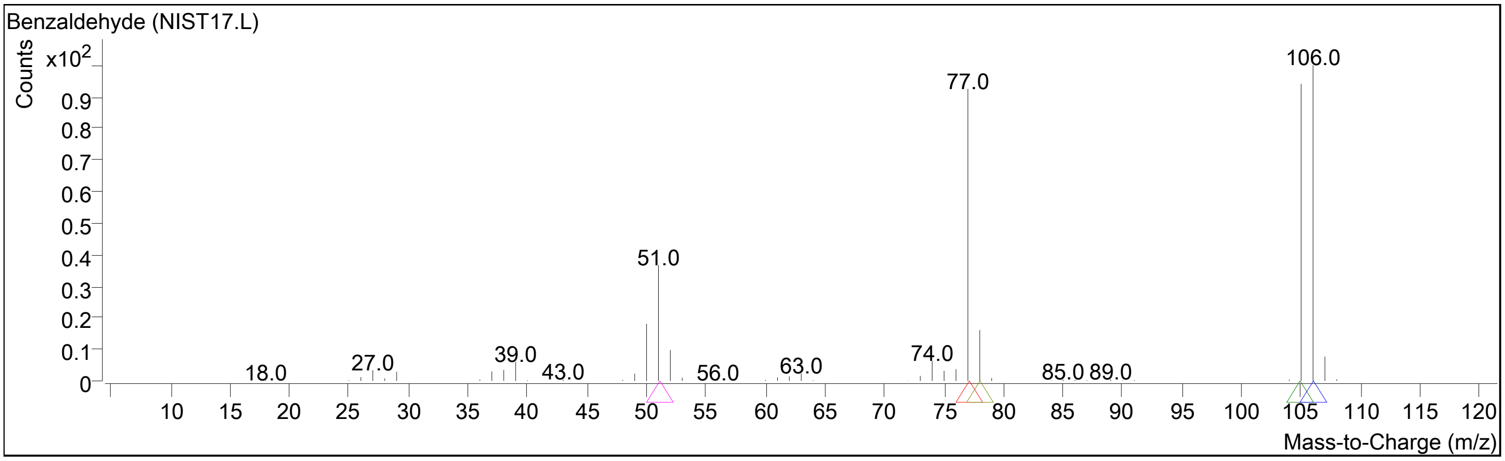

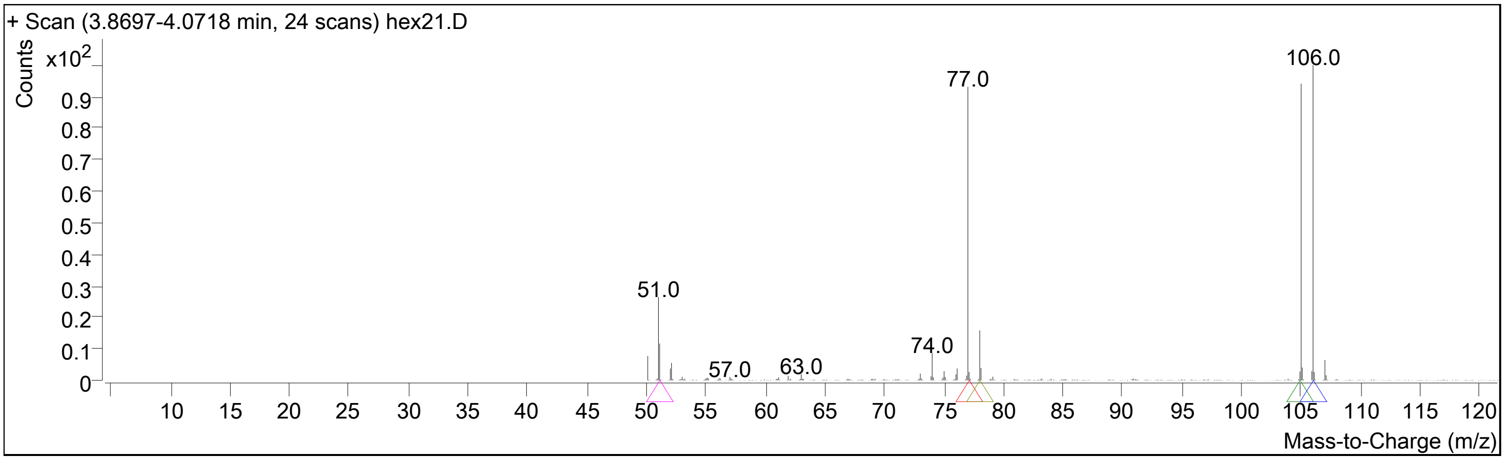

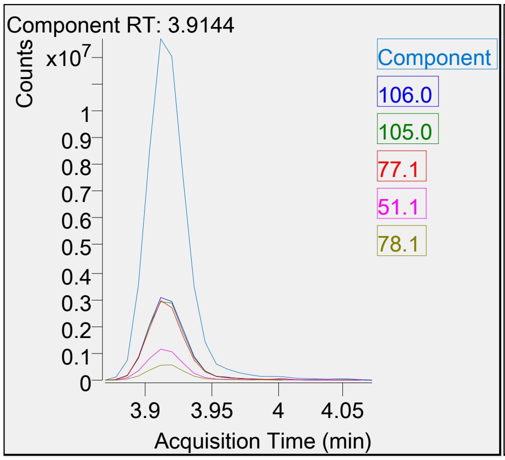

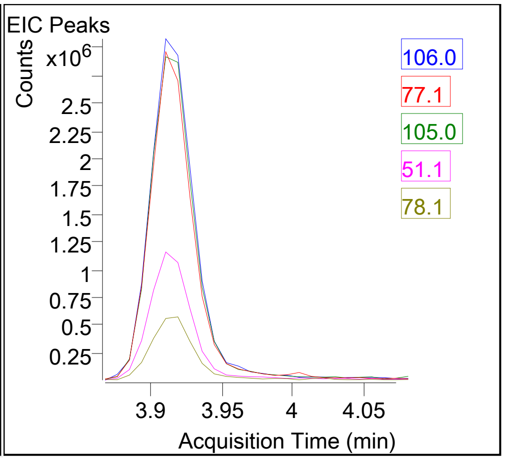

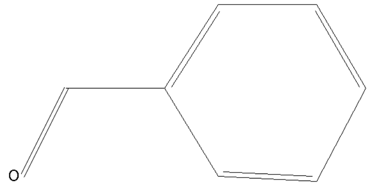

Unknown Analysis Report - Best Hits

Agilent Technologies

Component RT	Compound Name	CAS#	Formula	Area	Match Score	Est. Conc.
4.2899	Benzyl chloride	100-44-7	C7H7Cl	116515947	98.2	

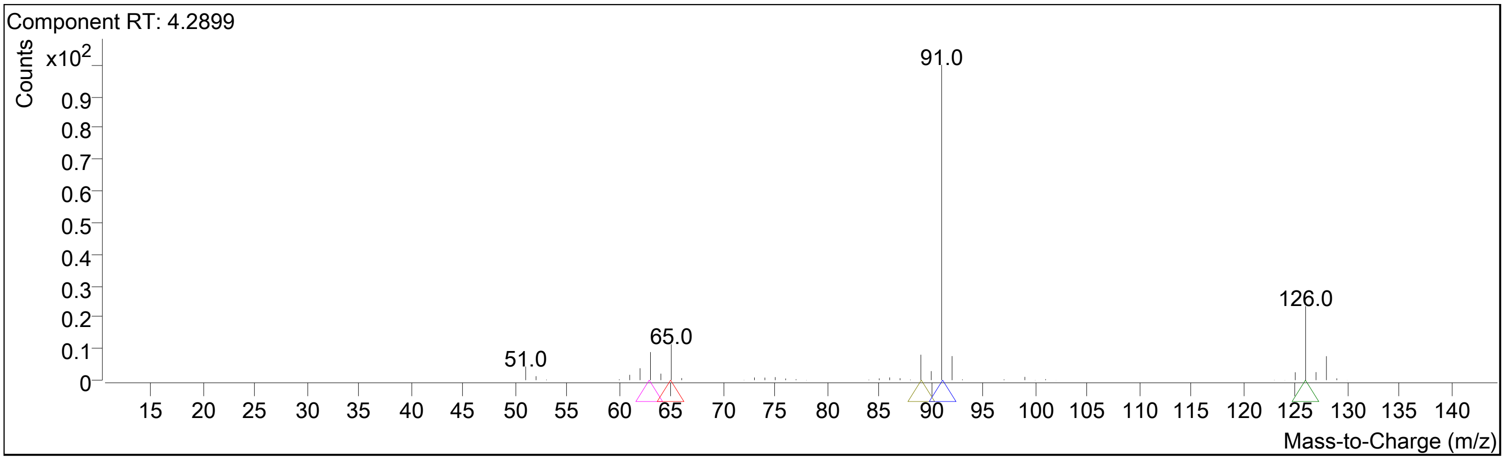

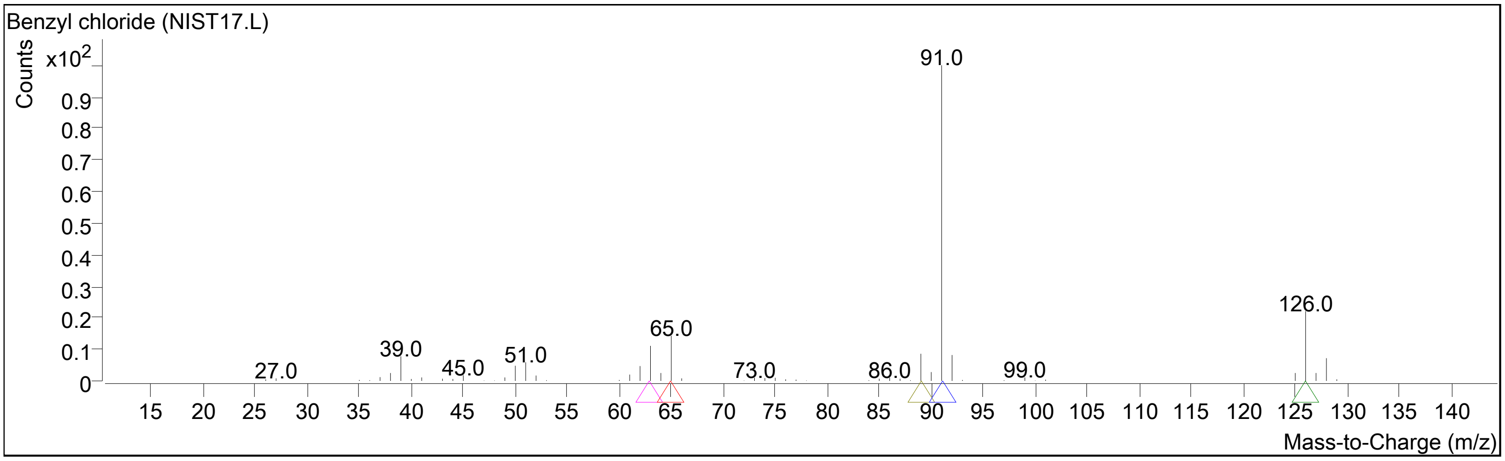

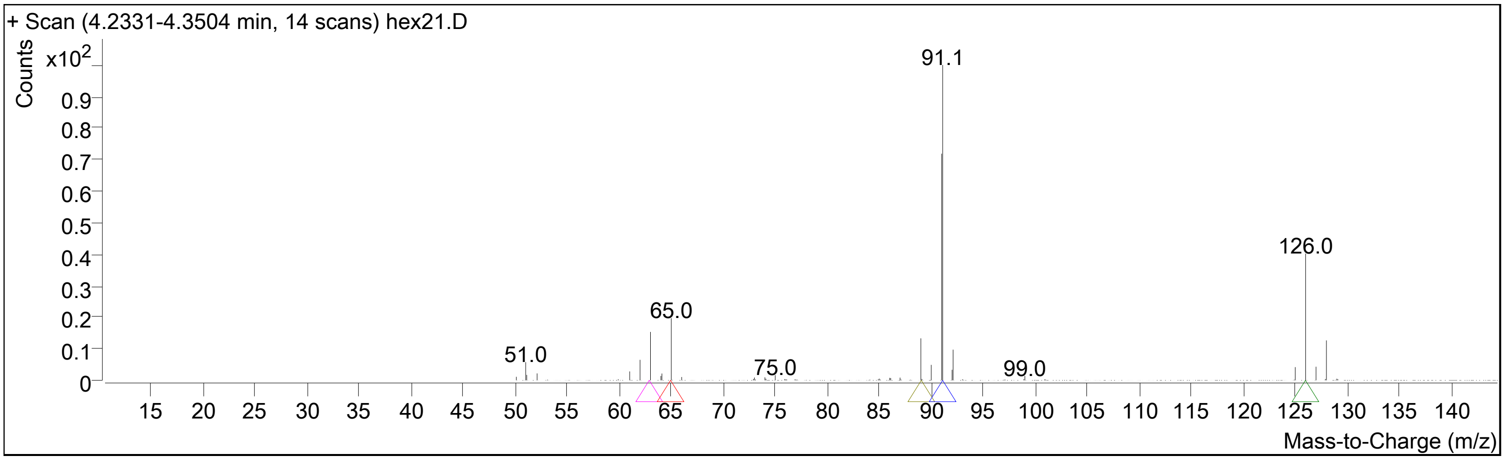

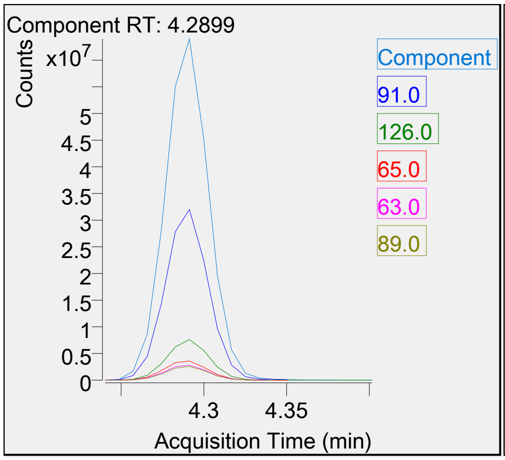

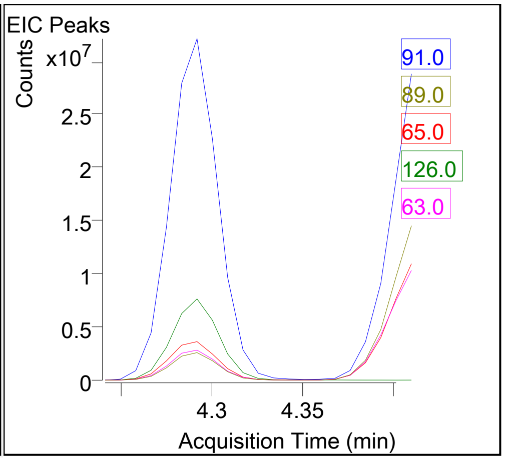

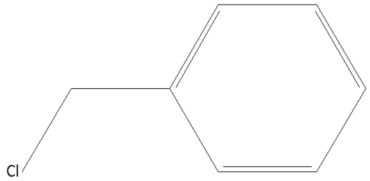

Unknown Analysis Report - Best Hits

Component RT	Compound Name	CAS#	Formula	Area	Match Score	Est. Conc.
4.4178	Benzyl alcohol	100-51-6	C7H8O	1961946901	99.6	

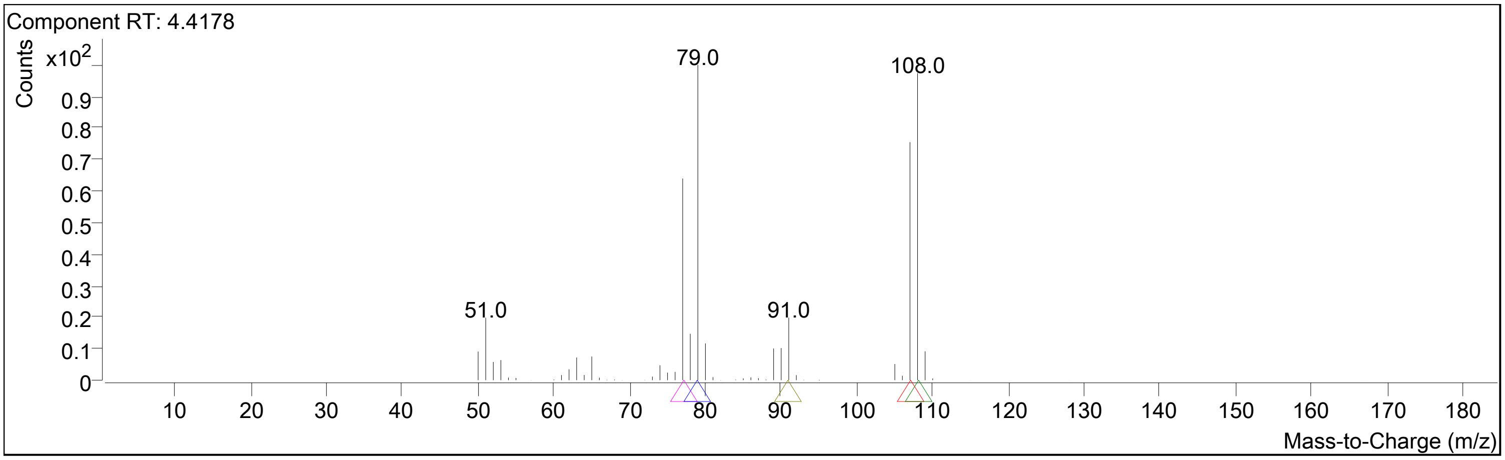

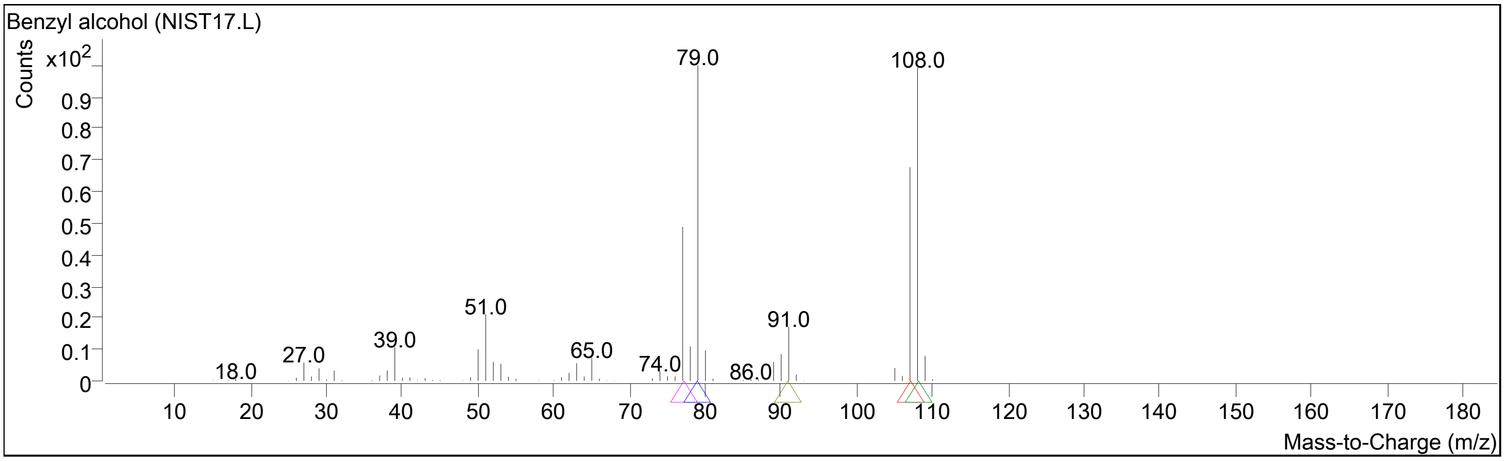

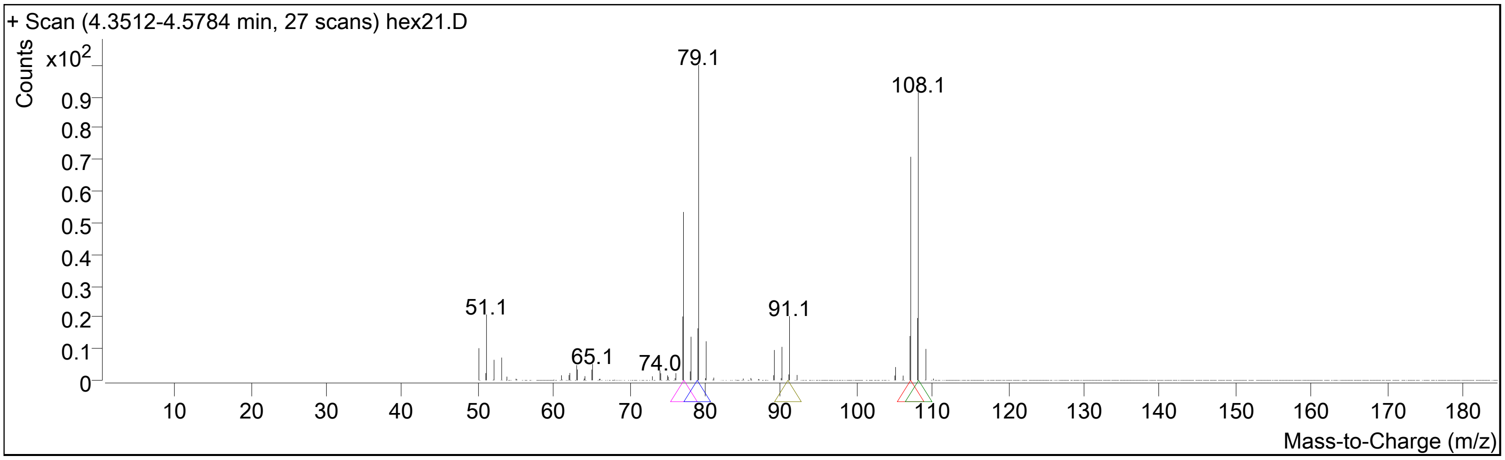

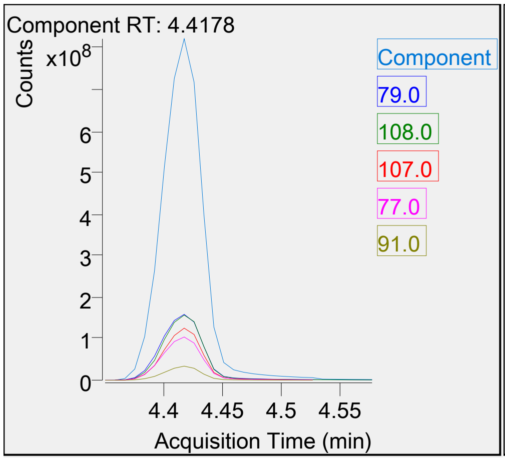

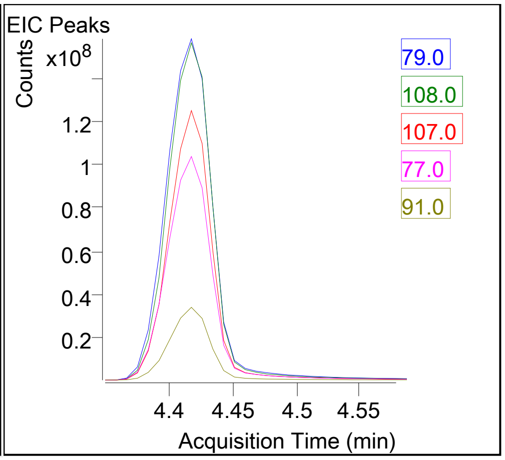

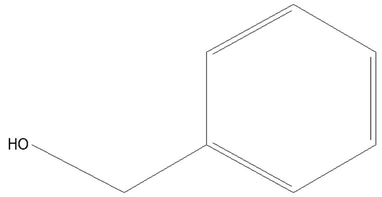

Agilent Technologies

Component RT	Compound Name	CAS#	Formula	Area	Match Score	Est. Conc.
8.3583	Benzene, 1,1'-[oxybis(methylene)]bis-	103-50-4	C14H14O	94125837	97.5	

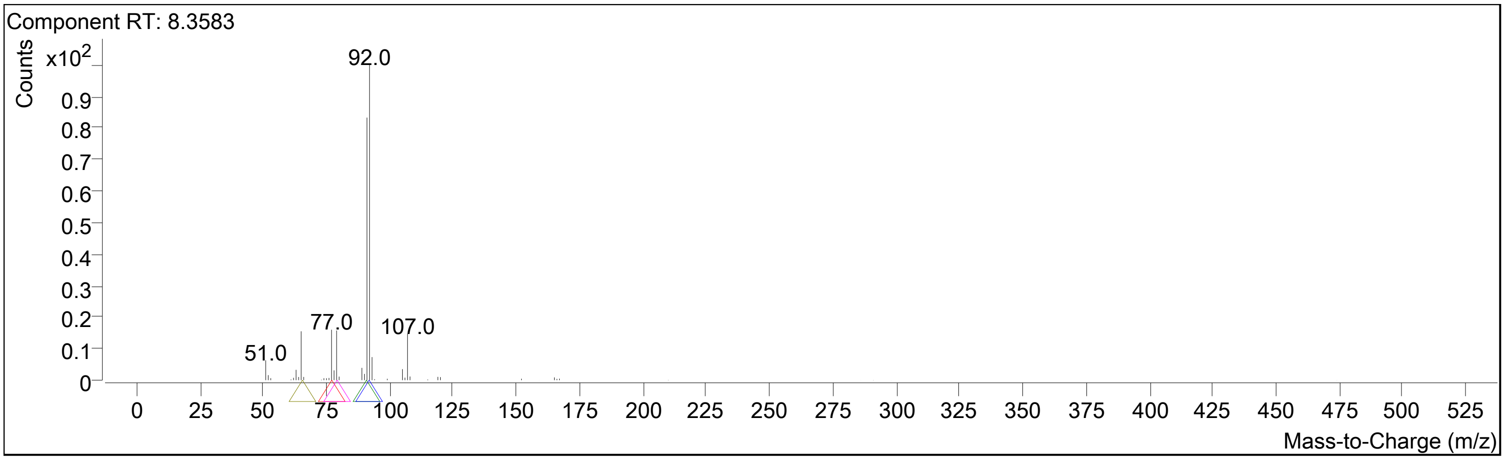

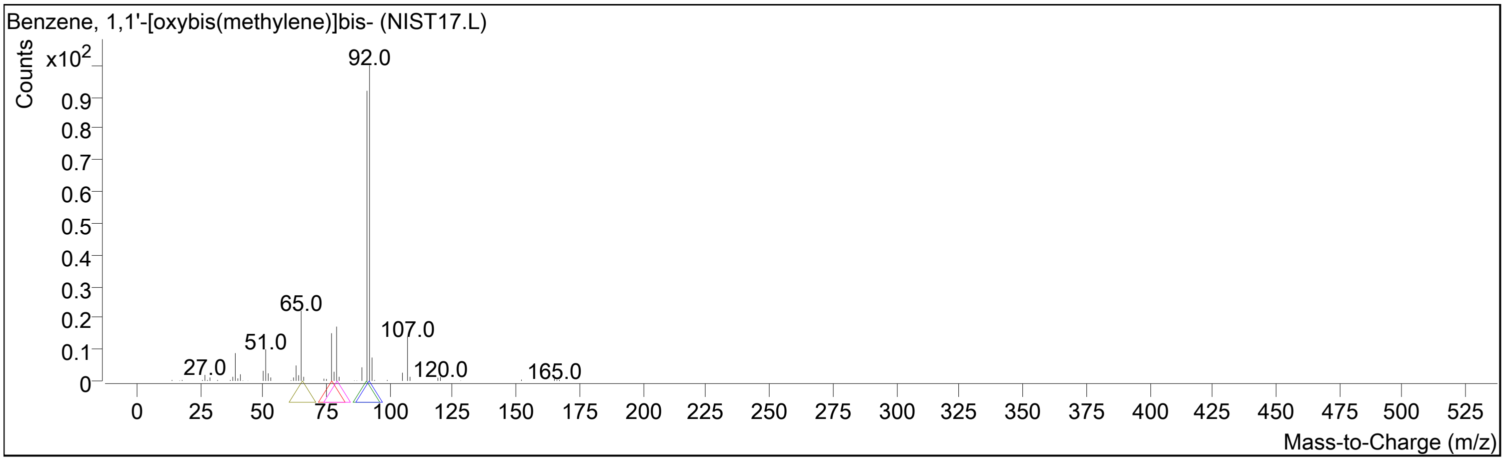

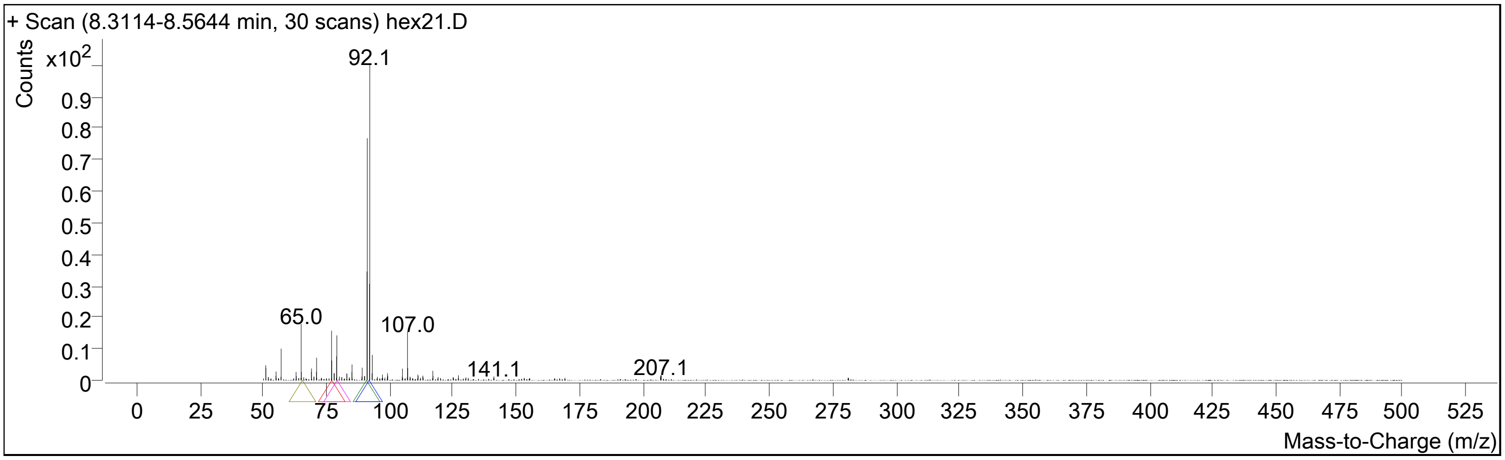

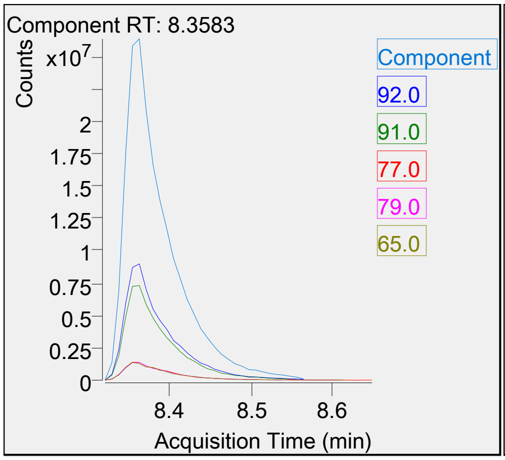

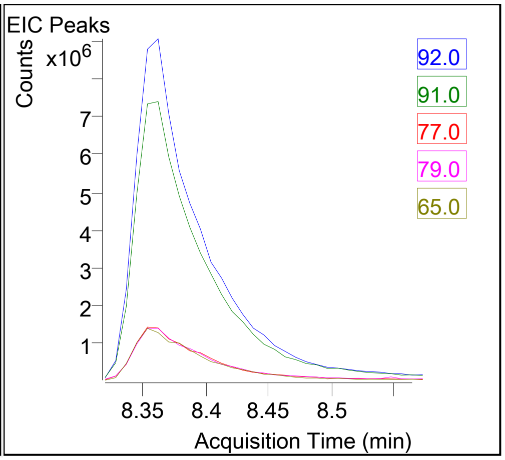

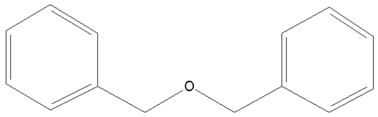

Unknown Analysis Report - Best Hits

Agilent Technologies

Component RT	Compound Name	CAS#	Formula	Area	Match Score	Est. Conc.
9.0259	Benzyl Benzoate	120-51-4	C14H12O2	20344136	93.6	

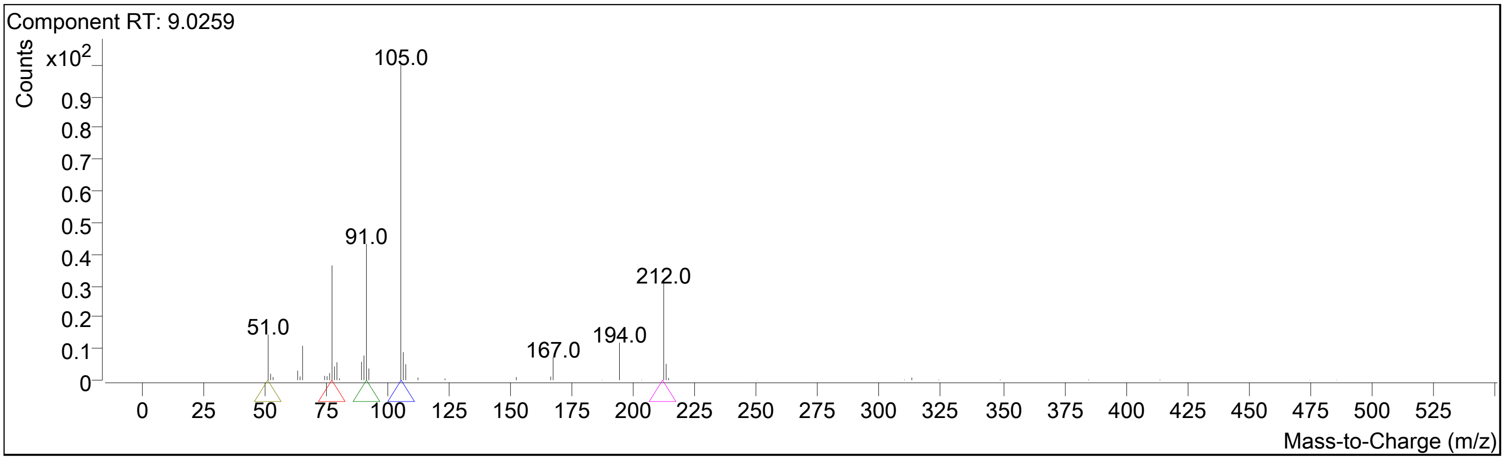

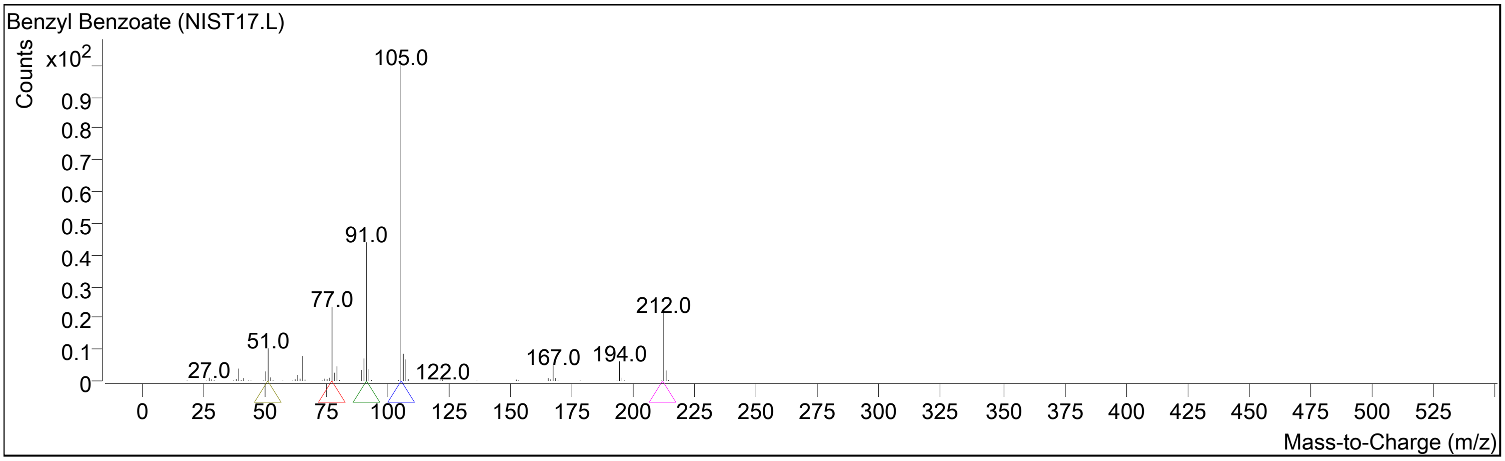

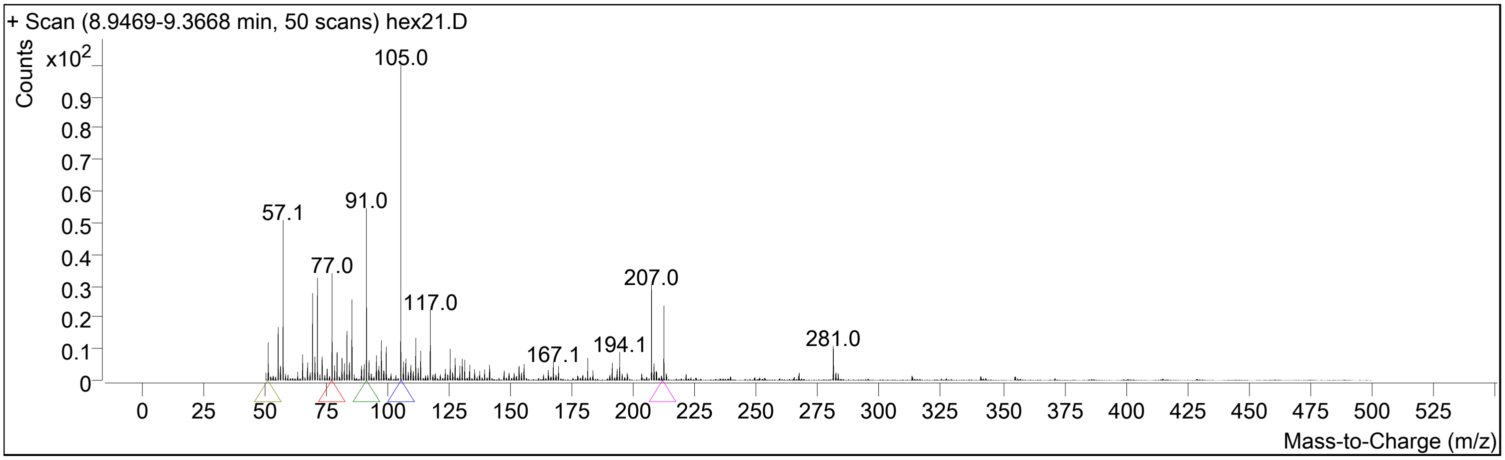

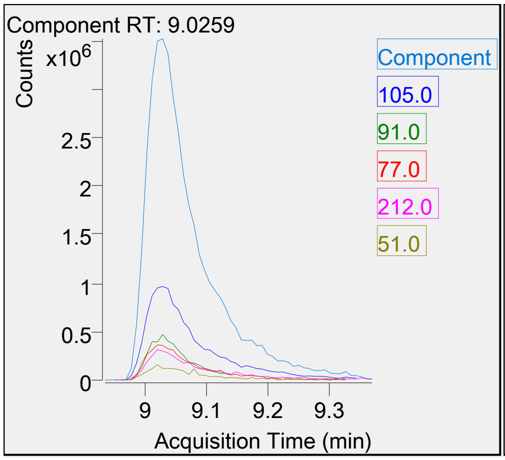

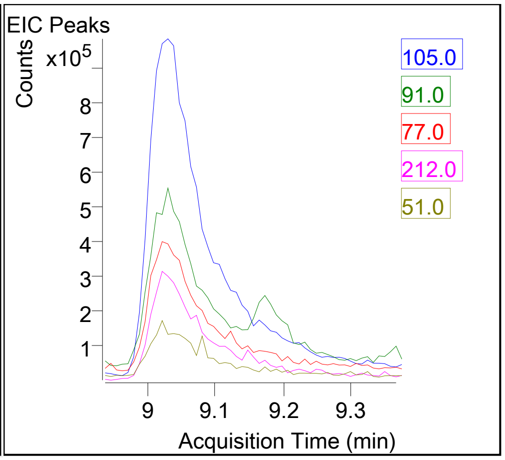

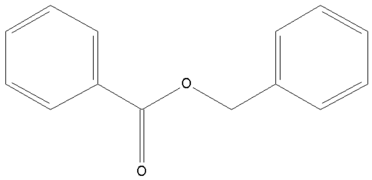

Agilent Technologies

**Batch Path**	E:\AS\dane\13_04_23		
**Analysis File Name**	test.uaf		
**Analyst Name**	Admin		
**Analysis Time**	4/19/2023 10:34:49 AM		
**Data File Name**	hex 22.D	**Data Path Name**	E:\AS\dane\13_04_23
**Sample Name**	hex 22	**Sample Type**	Sample
**Acq Method File**	alkbenz od 100 rampa 25stna min split 200	**Acq Method Path**	E:\AS\metody\
**Acq Time**	4/13/2023 1:46:43 PM	**Operator**	
**Instrument Name**	GCMSMS	**Dilution**	1

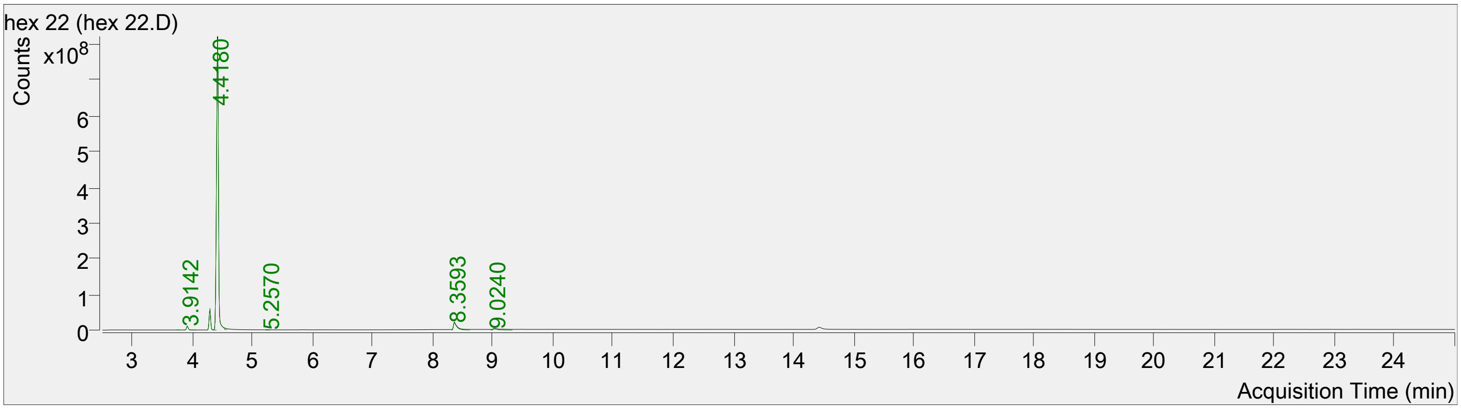

Component RT	Compound Name	CAS#	Formula	Area	Match Score	Est. Conc.
3.7638	Cyclotetrasiloxane, octamethyl-	556-67-2	C8H24O4Si4	509499	96.4	
3.9142	Benzaldehyde	100-52-7	C7H6O	22048593	99.2	
4.2900	Benzyl chloride	100-44-7	C7H7Cl	103967027	99.1	
4.4180	Benzyl alcohol	100-51-6	C7H8O	1898958923	99.3	
5.2570	Benzene, (dichloromethyl)-	98-87-3	C7H6Cl2	320232	90.0	
8.3593	Benzene, 1,1'-[oxybis(methylene)]bis-	103-50-4	C14H14O	81678499	98.0	
9.0240	Benzyl Benzoate	120-51-4	C14H12O2	15168710	95.4	

Unknown Analysis Report - Best Hits

Agilent Technologies

Component RT	Compound Name	CAS#	Formula	Area	Match Score	Est. Conc.
3.7638	Cyclotetrasiloxane, octamethyl-	556-67-2	C8H24O4Si4	509499	96.4	

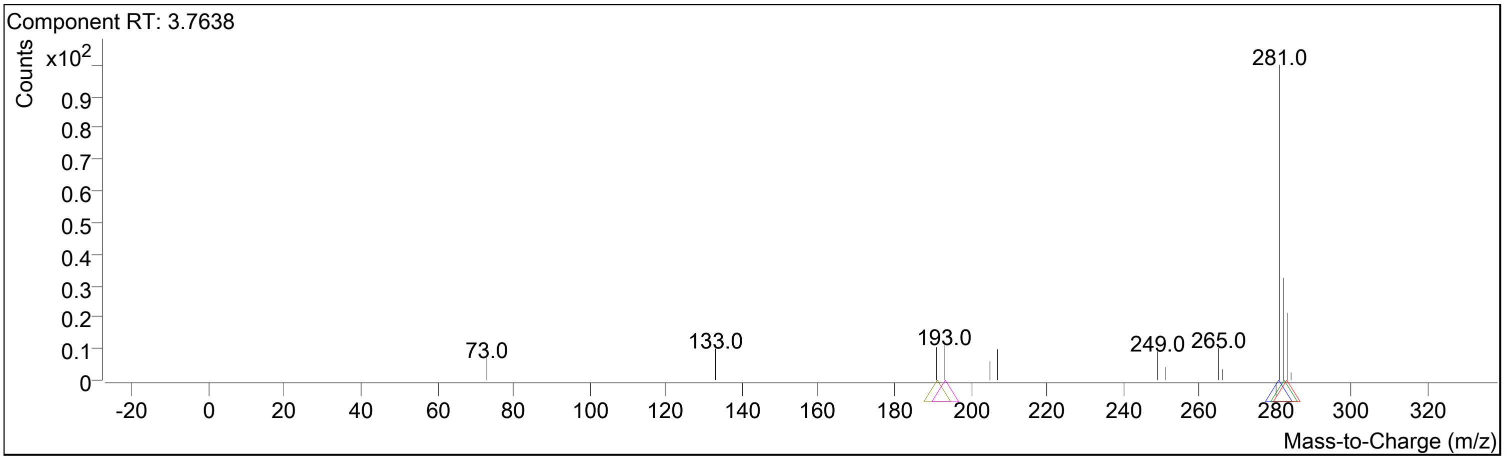

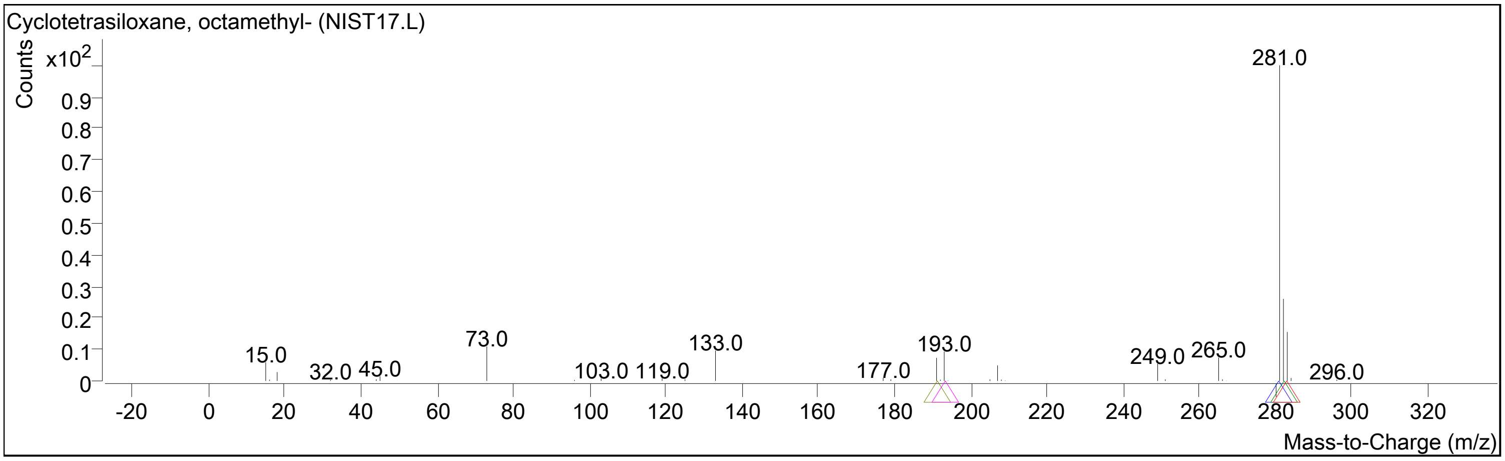

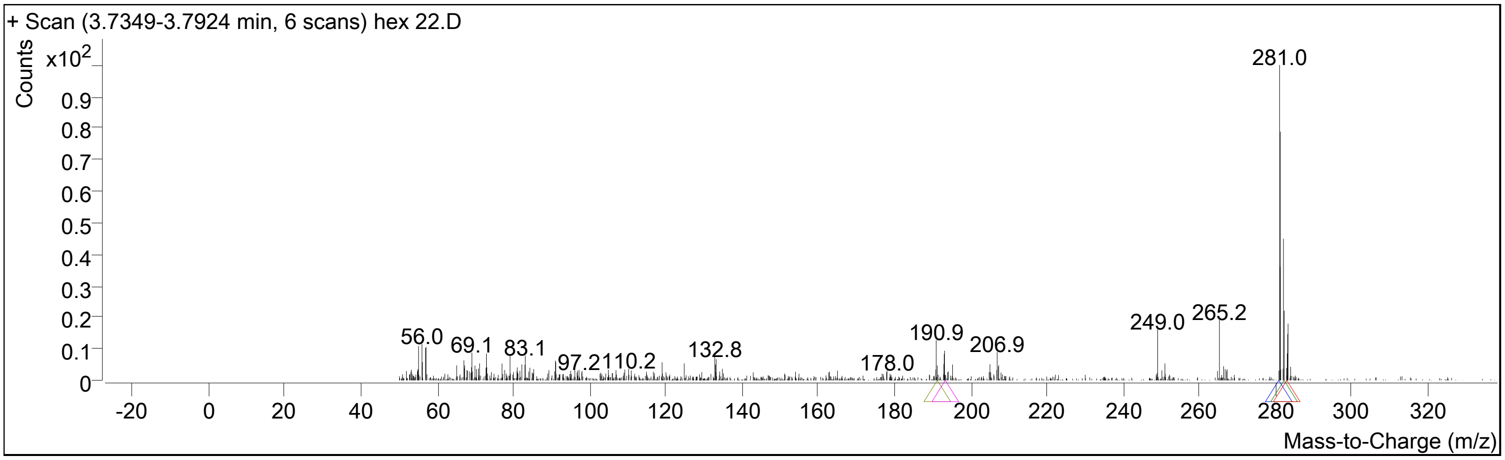

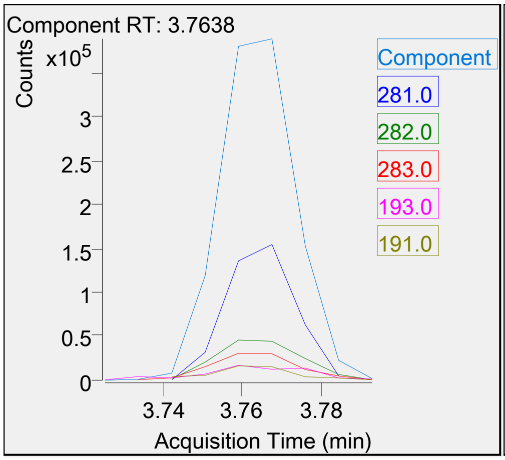

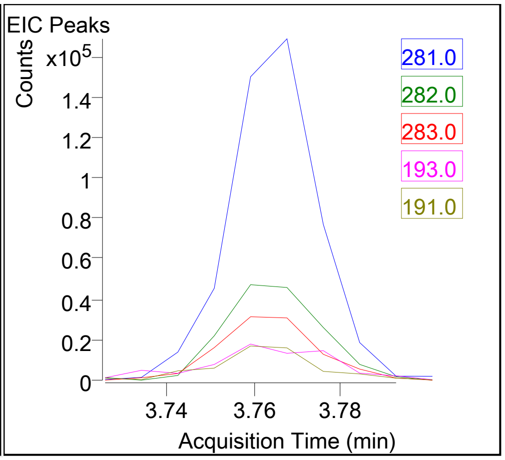

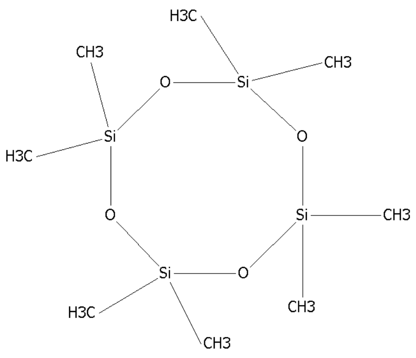

Unknown Analysis Report - Best Hits

Agilent Technologies

Component RT	Compound Name	CAS#	Formula	Area	Match Score	Est. Conc.
3.9142	Benzaldehyde	100-52-7	C7H6O	22048593	99.2	

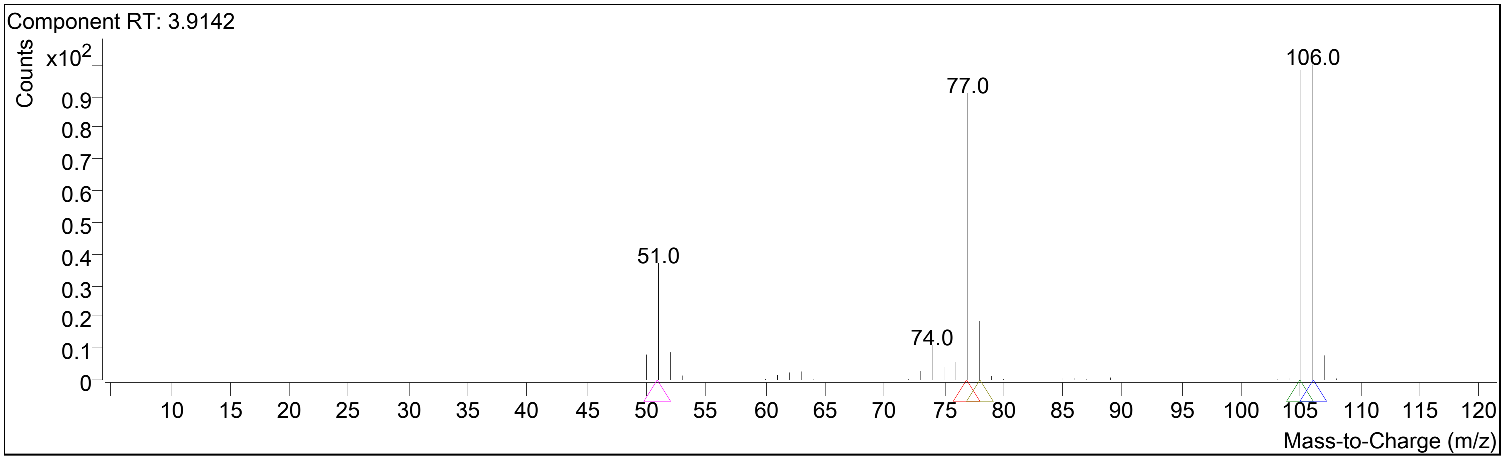

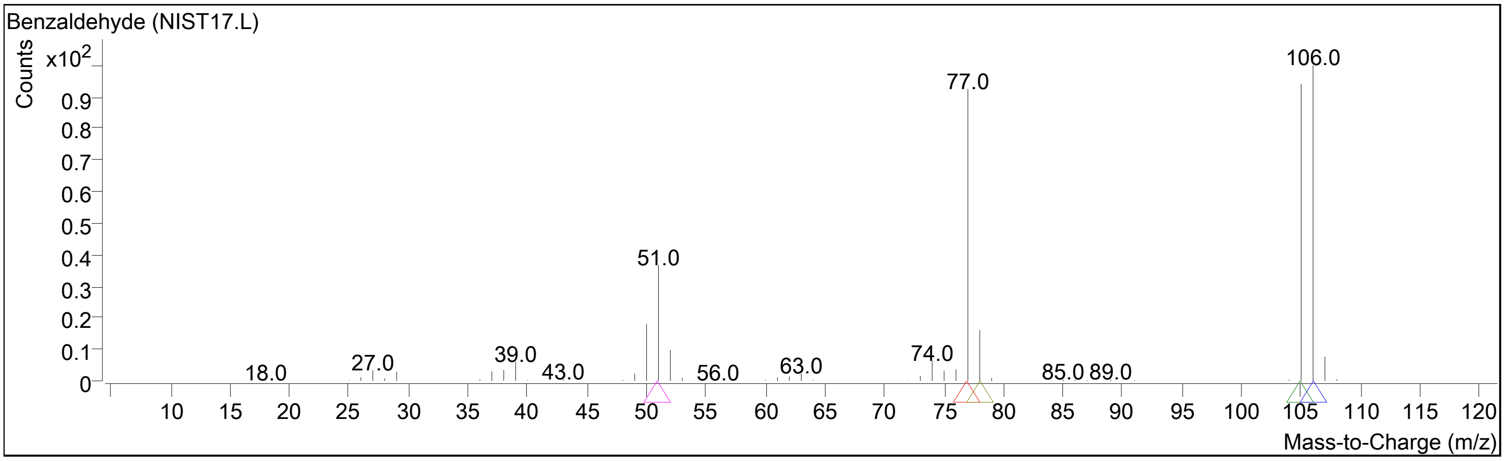

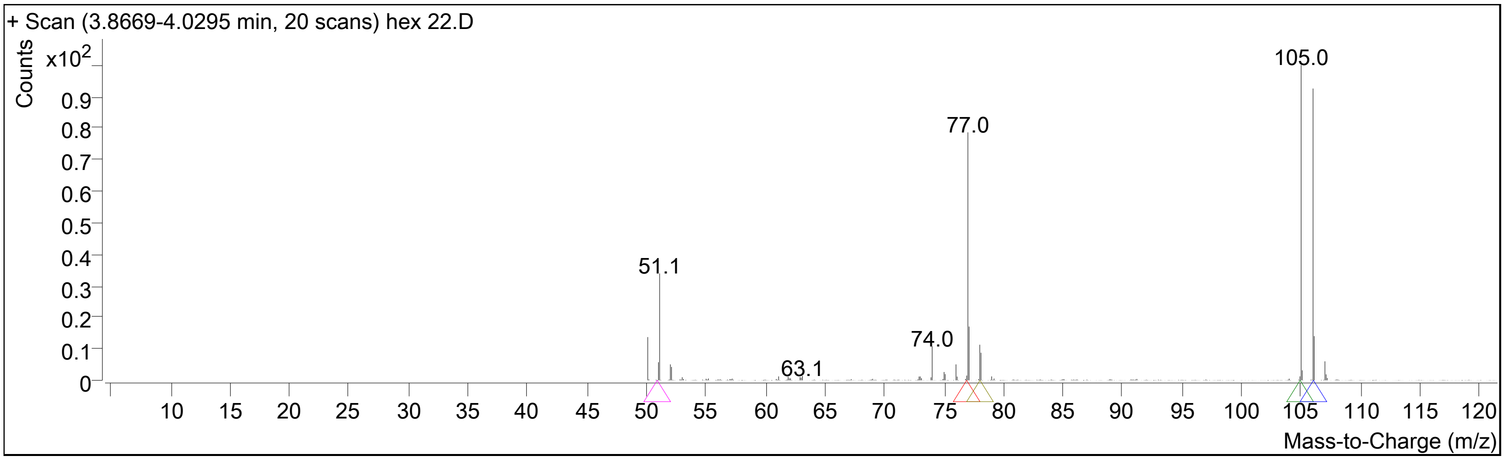

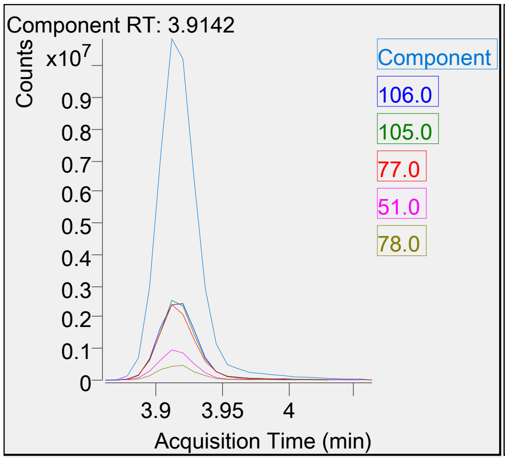

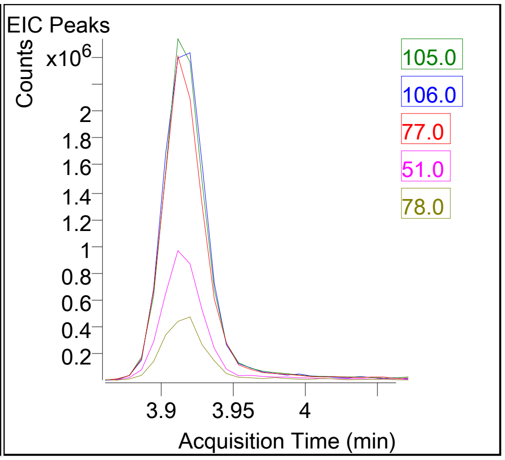

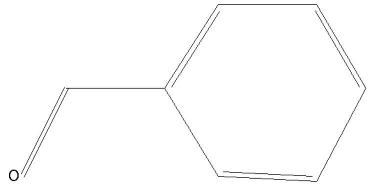

Unknown Analysis Report - Best Hits

Component RT	Compound Name	CAS#	Formula	Area	Match Score	Est. Conc.
4.2900	Benzyl chloride	100-44-7	C7H7Cl	103967027	99.1	

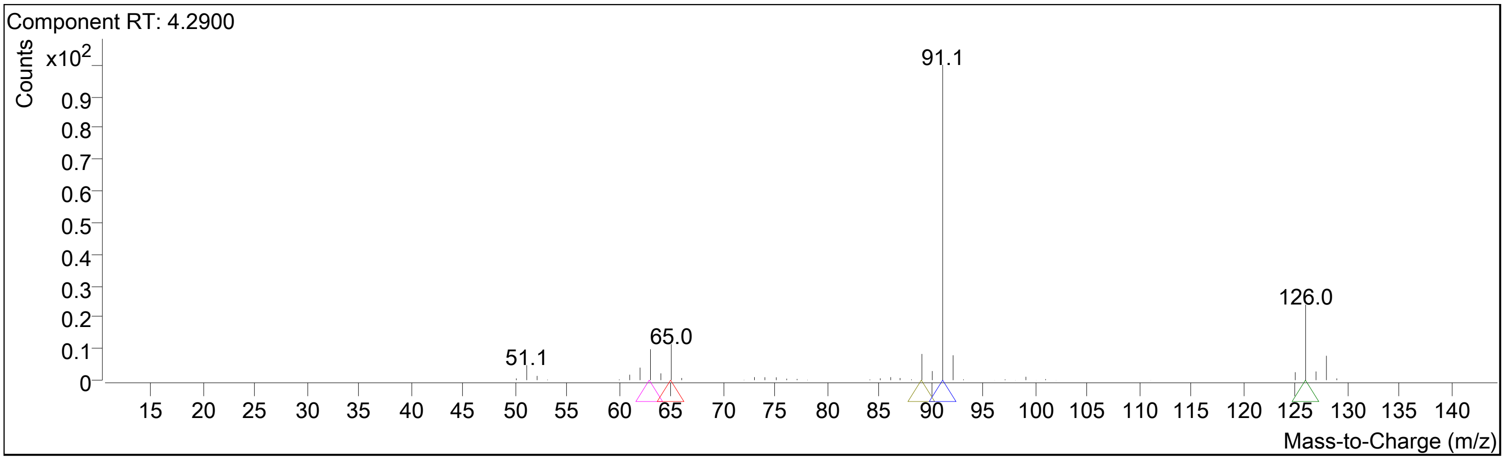

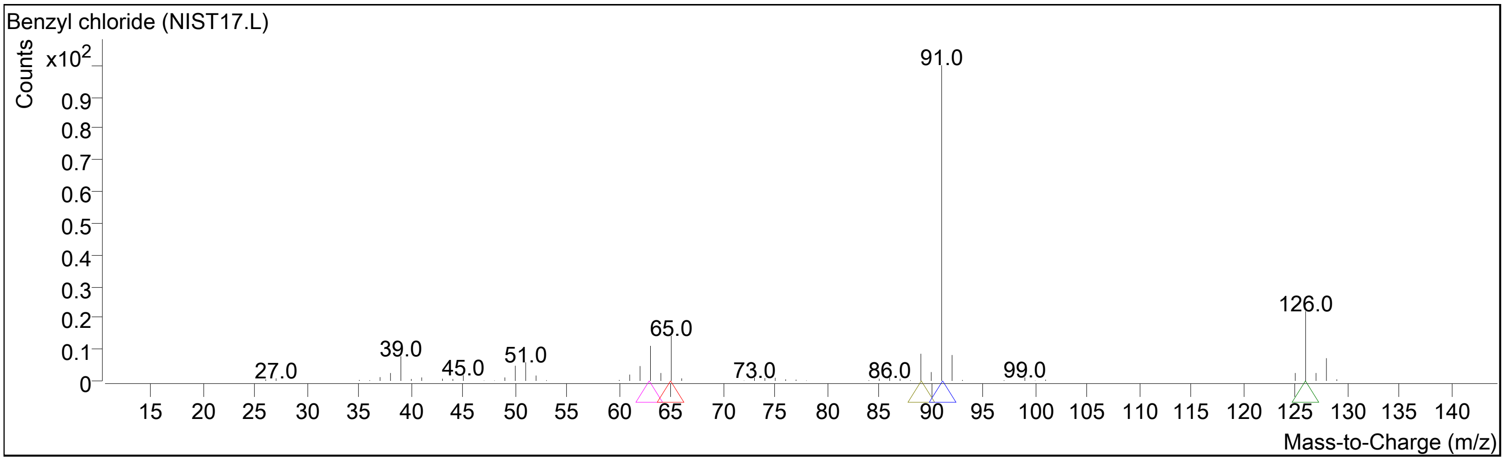

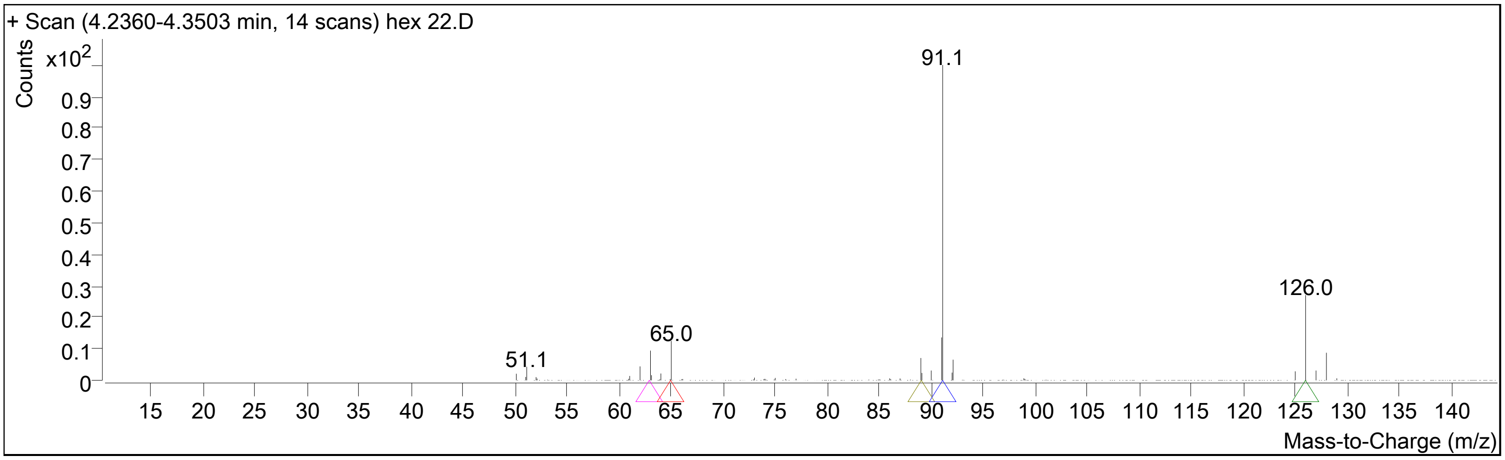

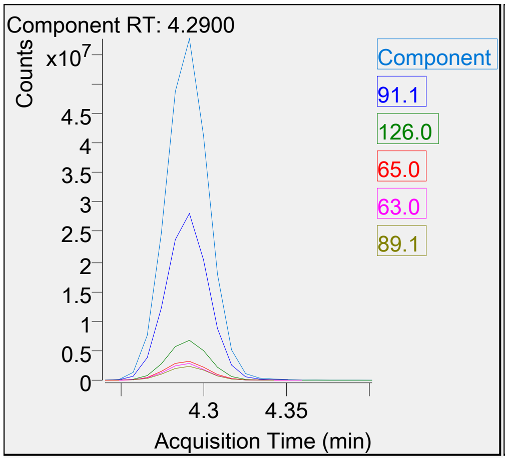

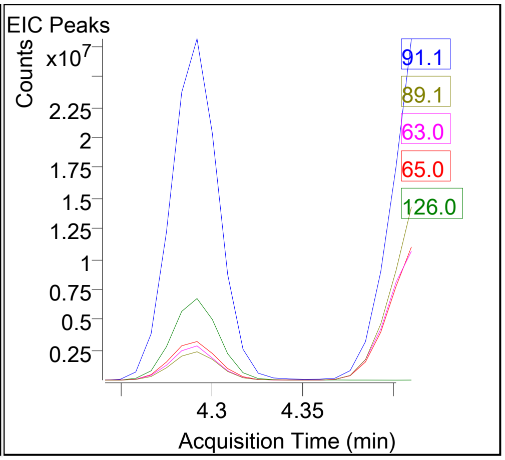

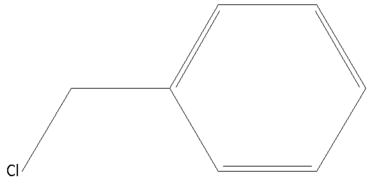

Unknown Analysis Report - Best Hits

Agilent Technologies

Component RT	Compound Name	CAS#	Formula	Area	Match Score	Est. Conc.
4.4180	Benzyl alcohol	100-51-6	C7H8O	1898958923	99.3	

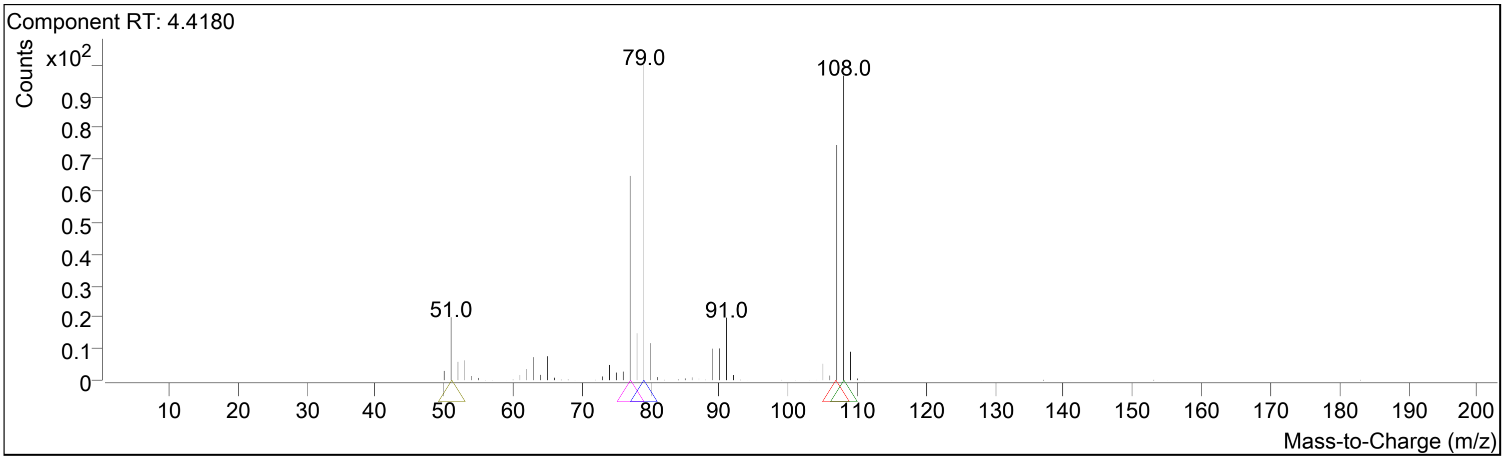

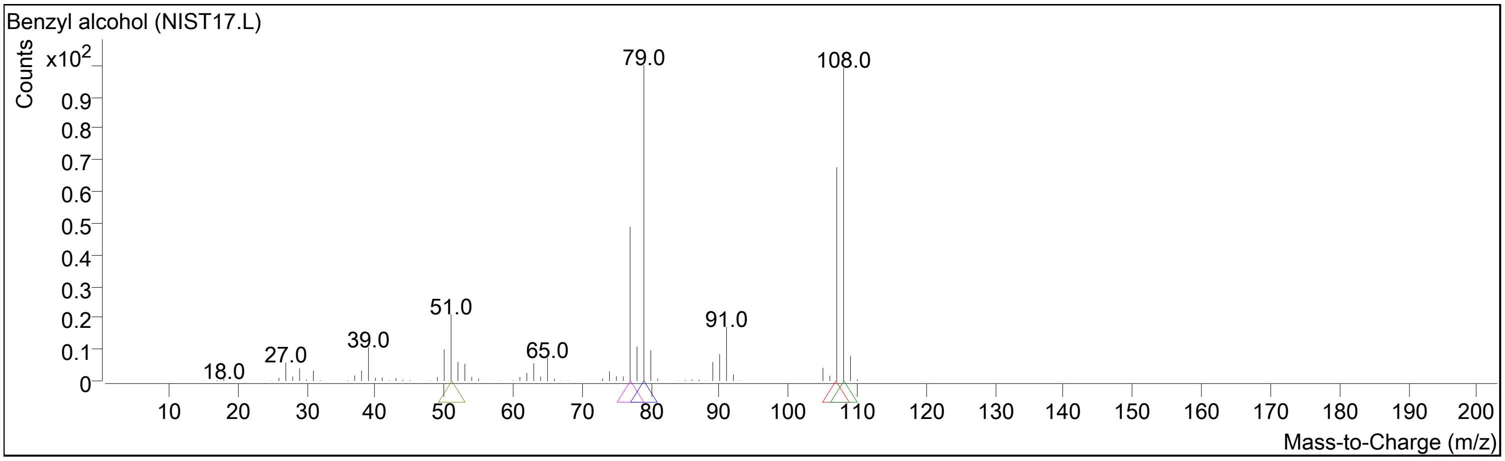

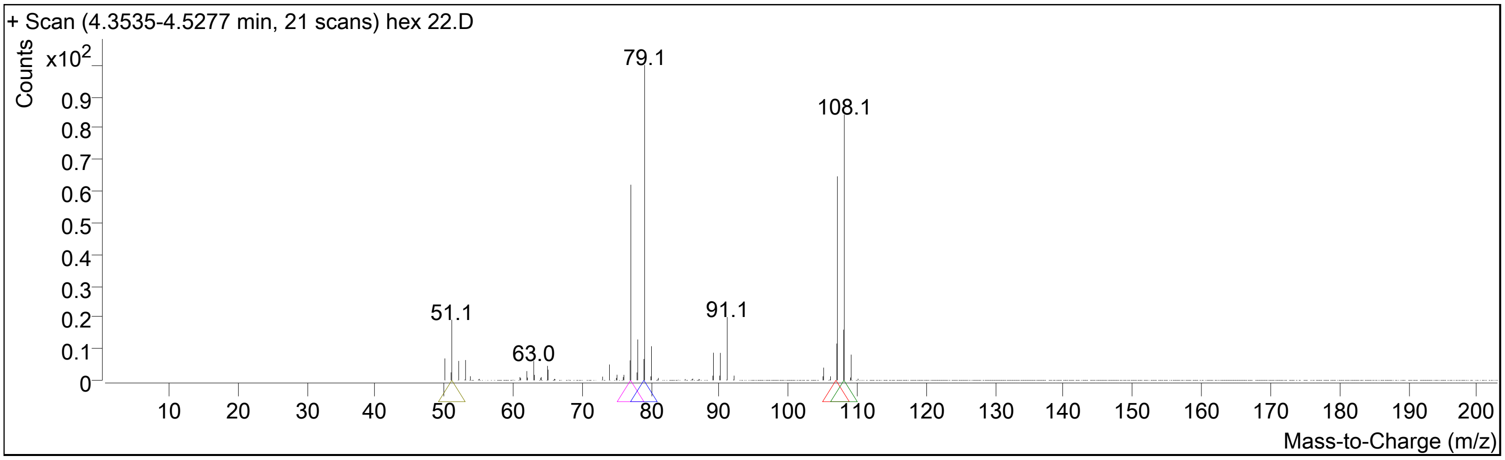

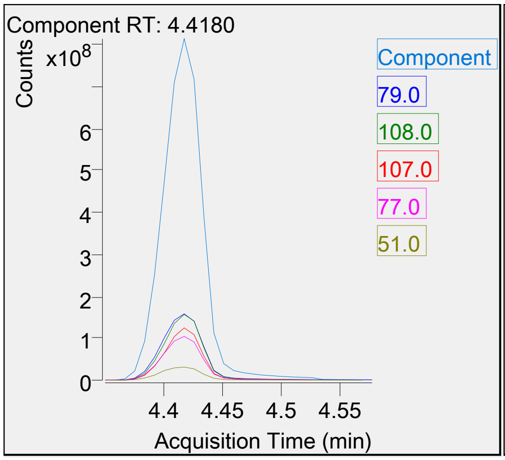

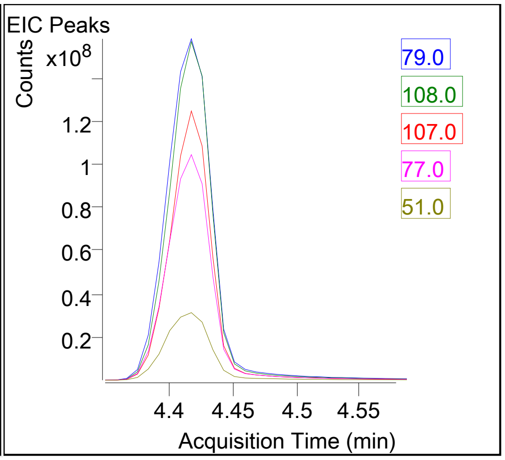

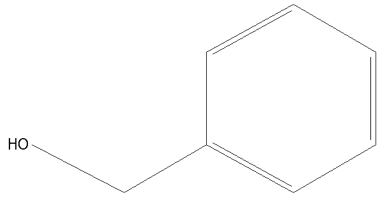

Unknown Analysis Report - Best Hits

Component RT	Compound Name	CAS#	Formula	Area	Match Score	Est. Conc.
5.2570	Benzene, (dichloromethyl)-	98-87-3	C7H6Cl2	320232	90.0	

Component RT: 5.2570

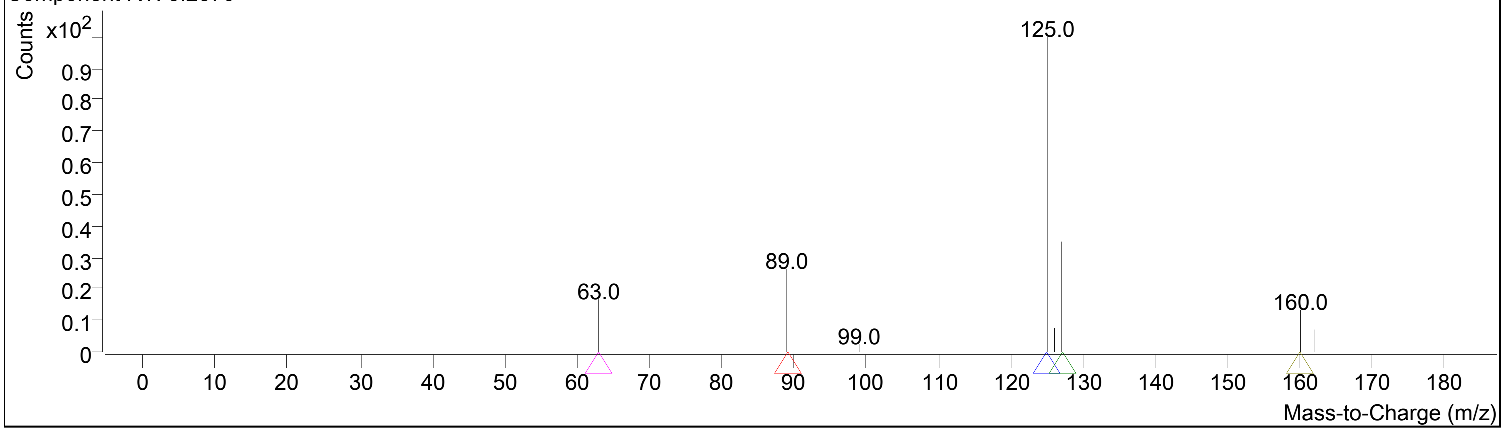

Benzene, (dichloromethyl)- (NIST17.L)

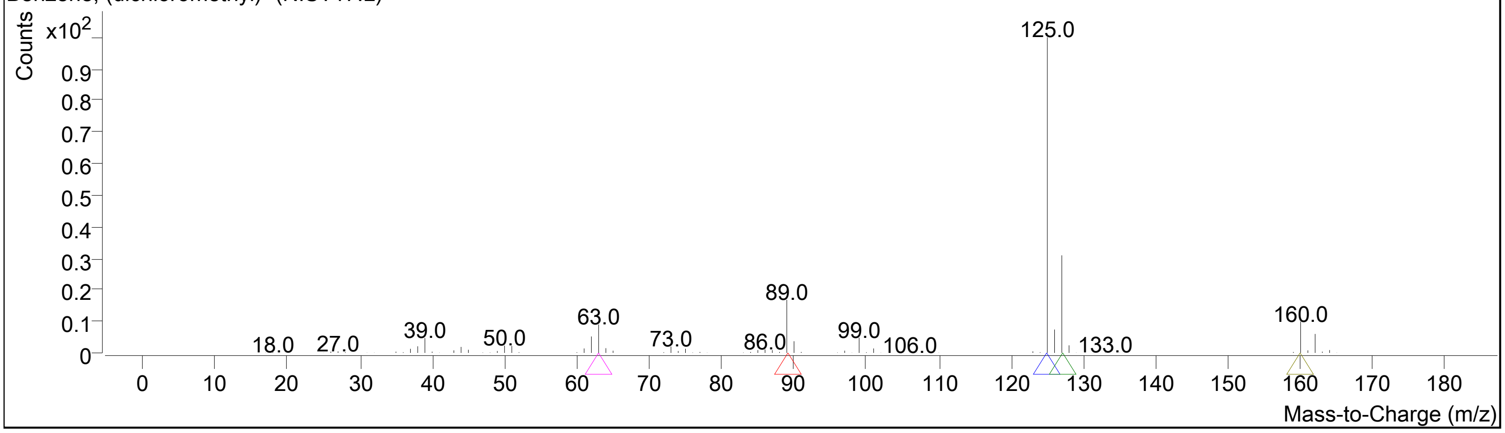

+ Scan (5.2242-5.3036 min, 9 scans) hex 22.D

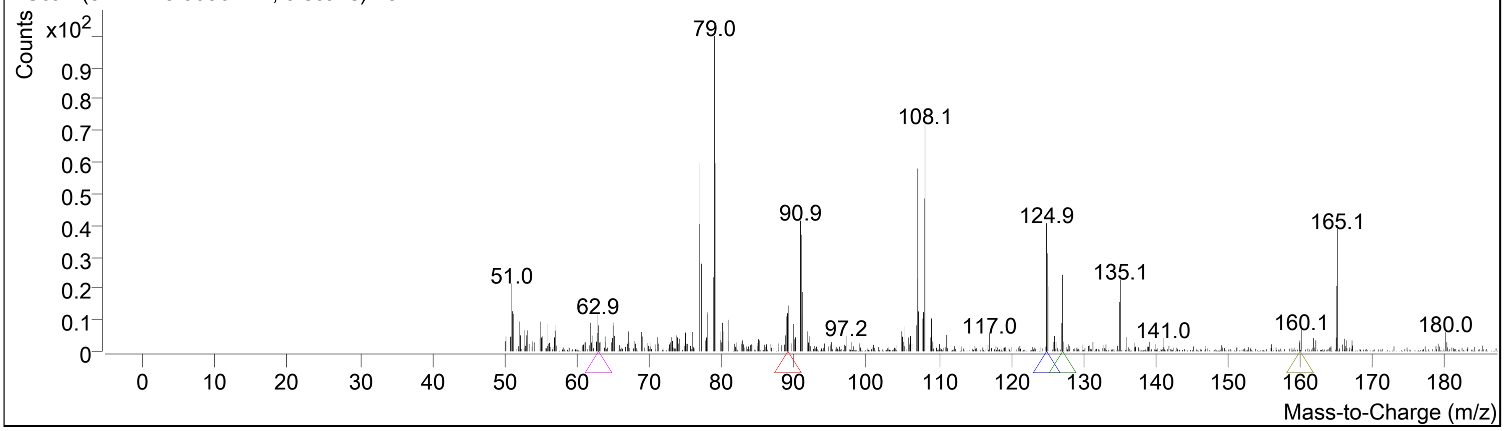

Component RT: 5.2570

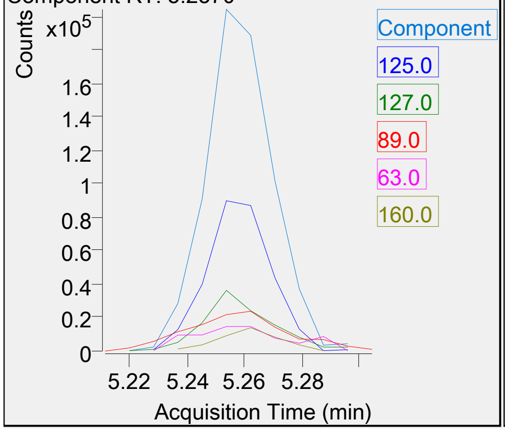

EIC Peaks

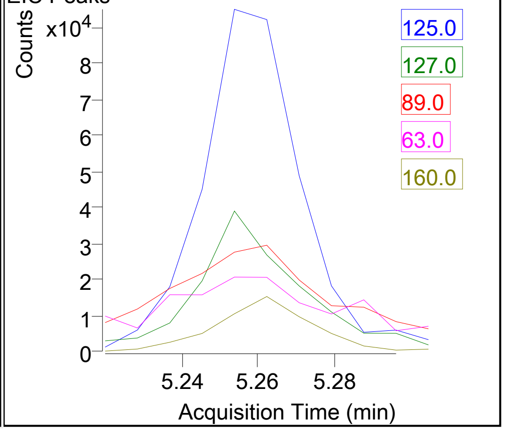

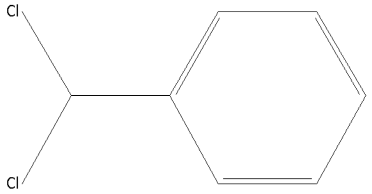

 Generated at 10:36 AM on 4/19/2023

Component RT	Compound Name	CAS#	Formula	Area	Match Score	Est. Conc.
8.3593	Benzene, 1,1'-[oxybis(methylene)]bis-	103-50-4	C14H14O	81678499	98.0	

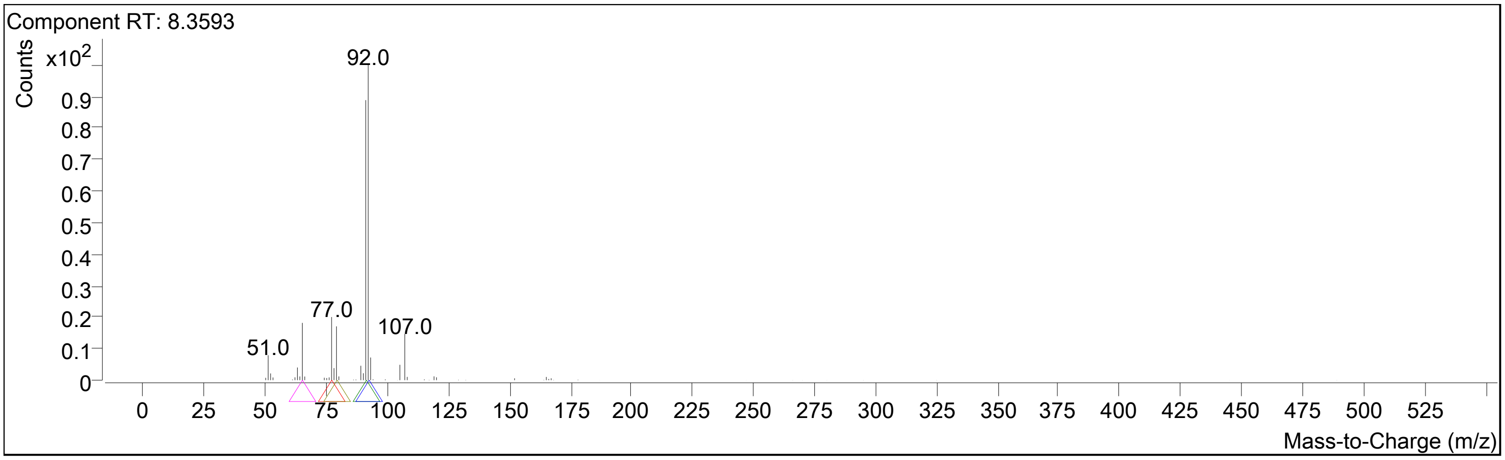

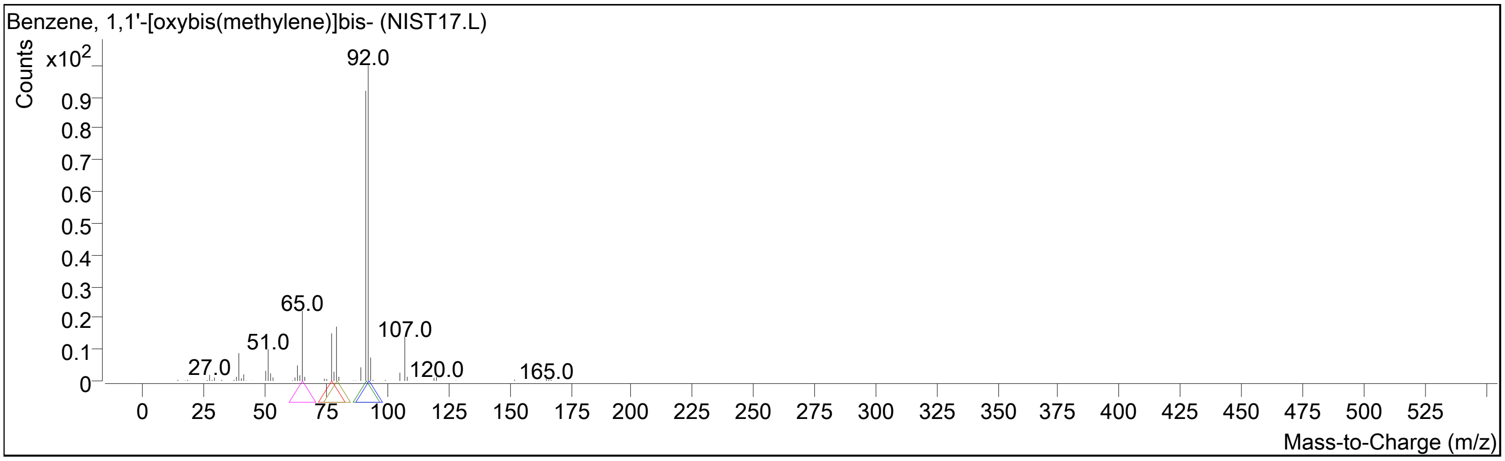

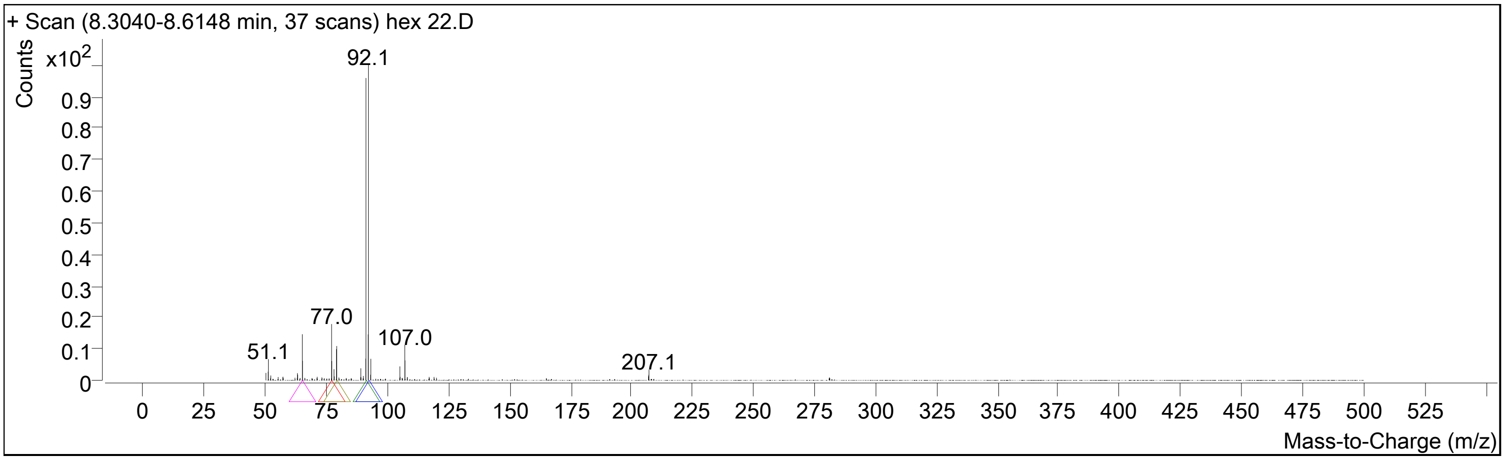

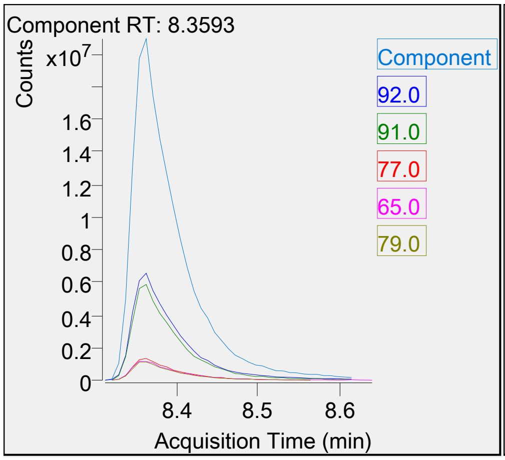

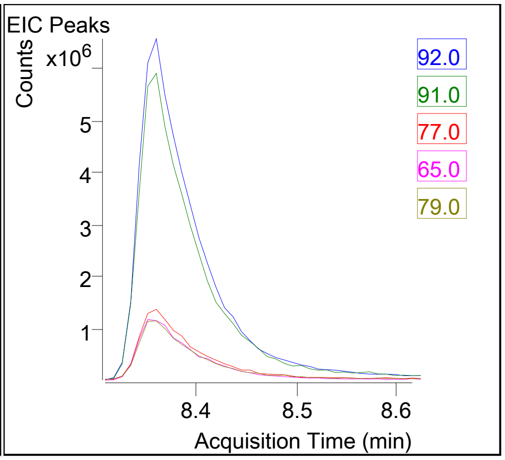

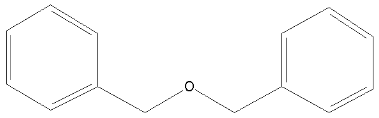

Unknown Analysis Report - Best Hits

Agilent Technologies

Component RT	Compound Name	CAS#	Formula	Area	Match Score	Est. Conc.
9.0240	Benzyl Benzoate	120-51-4	C14H12O2	15168710	95.4	

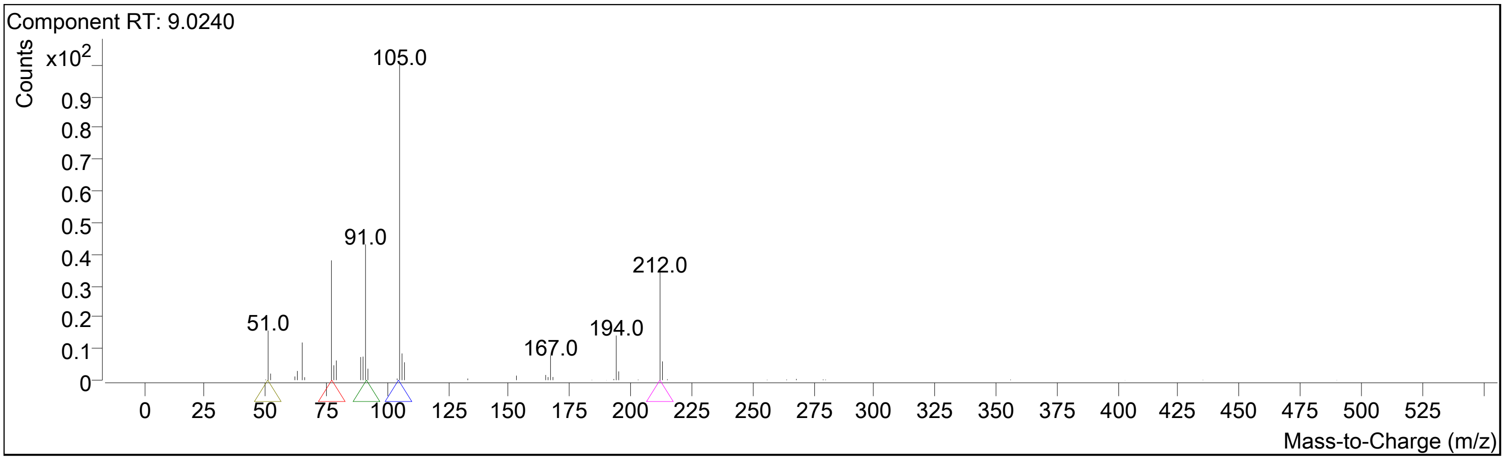

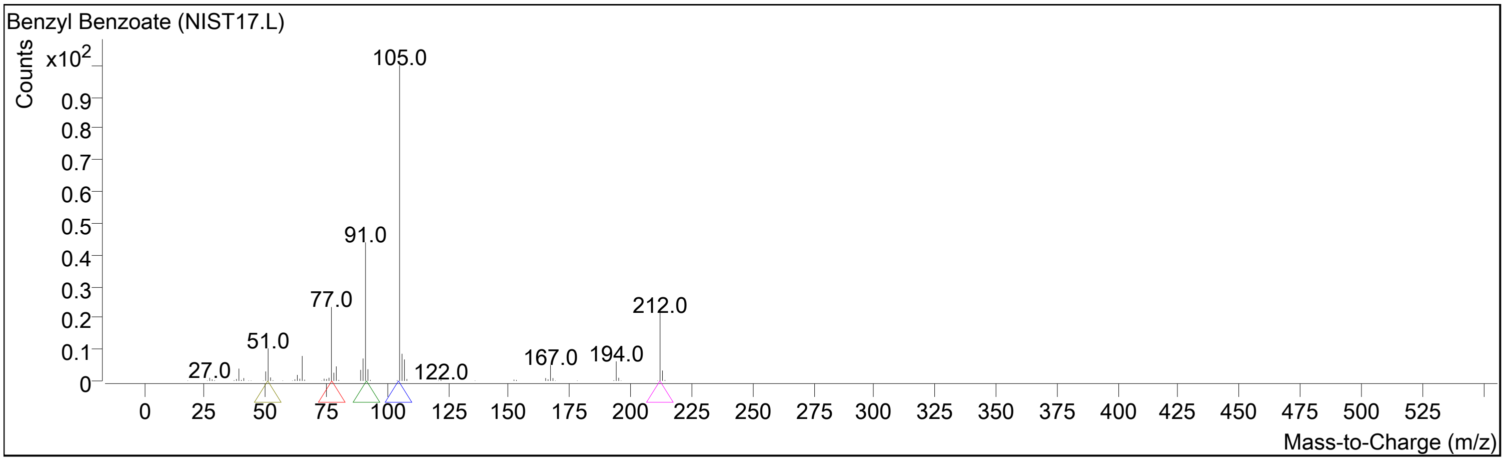

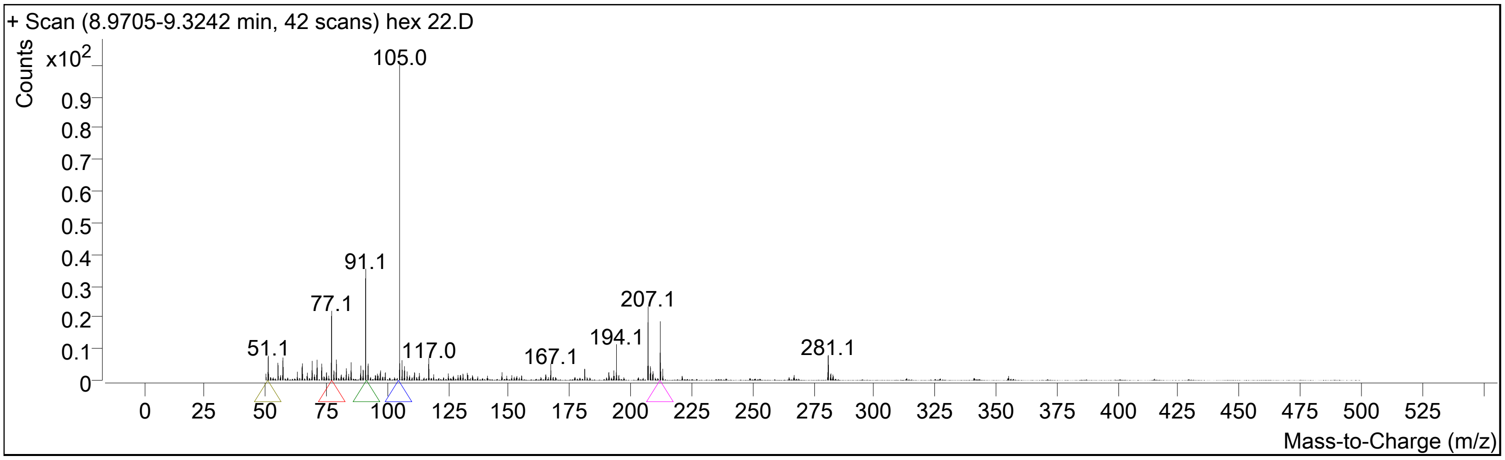

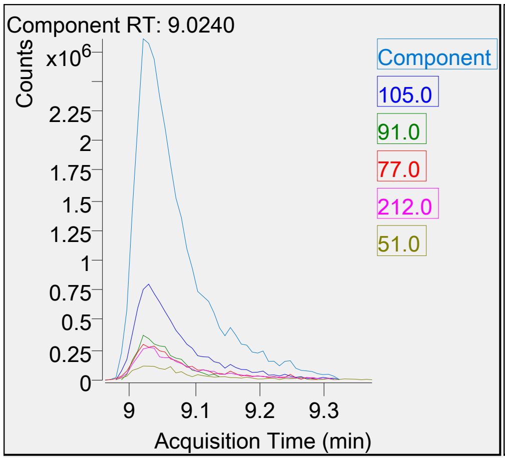

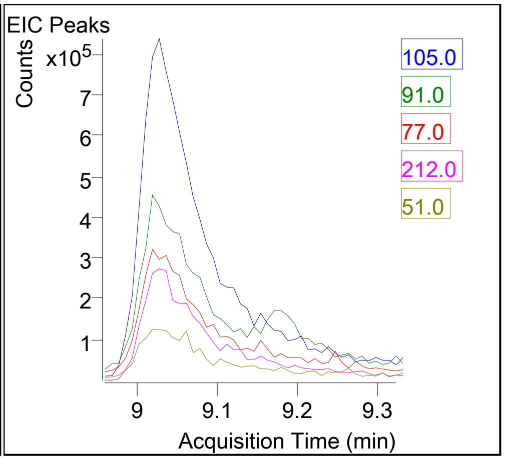

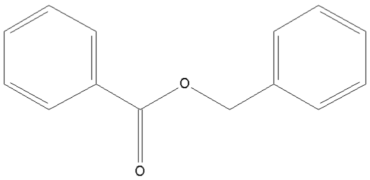

 Generated at 10:36 AM on 4/19/2023
